# Healthcare workers’ knowledge, attitudes and behaviours with respect to antibiotics, antibiotic use and antibiotic resistance across 30 EU/EEA countries in 2019

**DOI:** 10.2807/1560-7917.ES.2021.26.12.1900633

**Published:** 2021-03-25

**Authors:** Diane Ashiru-Oredope, Susan Hopkins, Sagar Vasandani, Eno Umoh, Olaolu Oloyede, Andrea Nilsson, John Kinsman, Linda Elsert, Dominique L Monnet, the #ECDCAntibioticSurvey Project Advisory Group, #ECDCAntibioticSurvey Project Advisory Group, #ECDCAntibioticSurvey Project Advisory Group, Reinhild Strauss, Vinciane Charlier, Samuel Coenen, Miranda Sertić, Marina Payerl-Pal, Linos Hadjihannas, Costas A. Constantinou, Barbora Macková, Lisa Bugge-Toft, Pille Märtin, Mailis Hansen, Outi Lyytikäinen, Jari Jalava, Anne Berger-Carbonne, Mélanie Colomb-Cotinat, Flora Kontopidou, Maria Foteinea, Martin Cormican, Audrey Lambourn, Francesca Furiozzi, Michela Sabbatucci, Elīna Dimiņa, Kate Vulāne, Virginija Kanapeckienė, Jolanta Kuklytė, Peter Zarb, Michael A. Borg, Renske Eilers, Harald Pors Muniz, Waleria Hryniewicz, Beata Mazińska, Duarte Pedro De Sousa Tavares, Livia Cioran, Alexandra Cucu, Eva Schreterova, Mitja Vrdelja, Maja Subelj, Rocío Bueno Parralo, Antonio López Navas, Agneta Andersson, Karin Carlin, Jacqui Reilly, Diane Ashiru-Oredope, Lea Pfefferle, Petr Horák, Steffen Amann, Andreas Trobisch, Lenneke Schrier, Tanguy Pinedo-Tora, Alyette Greiveldinger, Elena Carrara, Nico T. Mutters, Charles Price, Mathias Maucher, Michele Calabrò, Pascal Garel, Laurie Andrieu, Laura Alonso Irujo, María Santacreu García, Kitty Mohan, Sara Launio, Orsolya Réka Süli, Ivana Silva, Mervi Jokinen, Marta Simões, Ruben Viegas, Ann Marie Borg, Sascha Marschang, Céline Pulcini, Ber Oomen, Jeannette Verkerk, Ilaria Giannico, Paul Garassus, Roberto Bertollini, Melina Raso, Tímea Rezi-Kató, Jan De Belie, Ilaria Passarani, Jacques de Haller, Carole Rouaud, Jo Bosanquet, Wendy Nicholson, Cristiana Salvi

**Affiliations:** 1 Public Health England (PHE), London, United Kingdom; 2 European Centre for Disease Prevention and Control (ECDC), Stockholm, Sweden; 3 The members of the #ECDCAntibioticSurvey Project Advisory Group are listed at the end of the article

**Keywords:** behaviour change, COM-B, capability, opportunities, motivation, European Antibiotic Awareness Day (EAAD), Antibiotic Guardian, prescribers, prescribing

## Abstract

**Background:**

While several studies have assessed knowledge, attitudes and behaviours of the public, physicians and medical students in a number of EU/EEA countries with respect to antibiotic use and antibiotic resistance, there is a paucity of literature for other healthcare workers. This survey aimed to fill this gap.

**Methods:**

A 43-item online questionnaire was developed, validated and pilot-tested through a modified Delphi consensus process involving 87 Project Advisory Group (PAG) members, including national representatives and members of European health professional groups. The survey was distributed by the PAG and via social media to healthcare workers in 30 EU/EEA countries.

**Results:**

Respondents (n = 18,365) from 30 EU/EEA countries participated. Knowledge of antibiotics and antibiotic use was higher (97%) than knowledge of development and spread of antibiotic resistance (75%). Sixty percent of respondents stated they had received information on avoiding unnecessary prescribing, administering or dispensing of antibiotics. Among respondents who prescribed, administered or dispensed antibiotics, 55% had provided advice on prudent antibiotic use or management of infections to patients, but only 17% had given resources (leaflets or pamphlets). For community and hospital prescribers, fear of patient deterioration or complications was the most frequent reason (43%) for prescribing antibiotics that were considered unnecessary. Community prescribers were almost twice as likely as hospital prescribers to prescribe antibiotics due to time constraints or to maintain patient relationships.

**Conclusion:**

It is important to move from raising awareness about prudent antibiotic use and antibiotic resistance among healthcare workers to designing antimicrobial stewardship interventions aimed at changing relevant behaviours.

## Introduction

In Europe, 33,000 people die from infections with antibiotic-resistant bacteria each year. An estimated EUR 1.5 billion is spent annually on healthcare costs and loss of productivity due to antibiotic resistance [1,2]. The causes of misuse or overuse of antibiotics are multifactorial and include a lack of understanding, clarity and knowledge about antibiotics, antibiotic use and the development and spread of antibiotic resistance. In 2008, the European Centre for Disease Prevention and Control (ECDC) launched the European Antibiotic Awareness Day (EAAD), a European health initiative to raise awareness about the need for prudent use of antibiotics, targeting both the public and healthcare workers [3].

While previous multi-country and European Union (EU)-wide studies have focused on the public‘s [4-7], physicians’ and medical students’ understanding of antibiotics, antibiotic use and antibiotic resistance (in three to six countries) [8-13], there is a paucity of evidence for other healthcare workers or wider multi-country or multi-professional studies. This is important because all healthcare workers play a critical role in the use of antibiotics, from educating patients to minimising the spread of infection in healthcare settings, particularly when they are directly involved in the treatment of infections through prescribing, dispensing and administering of antibiotics [14,15].

This is a baseline study, which aims to: (i) assess the knowledge, attitudes and behaviours of healthcare workers in 30 EU/EEA (European Union/European Economic Area) countries with respect to antibiotics, antibiotic use and antibiotic resistance; (ii) provide a baseline dataset for designing and evaluating future policy, communication and educational interventions; and (iii) support the evaluation of awareness raising campaigns including EAAD.

## Methods

In October 2018, ECDC National Focal Points for Antimicrobial Resistance and National Focal Points for Communication from all EU countries, two EEA countries (Iceland and Norway), and selected European health professional organisations or groups, were invited to designate representatives to participate in the present study as members of a Project Advisory Group (PAG). The PAG comprised 87 individuals representing the EU/EEA countries and European professional organisations listed at the end of the article.

Many models of behaviour change have been used to understand and predict health behaviour. Examples include the theory of reasoned action, the health belief model, social learning theory, and the trans-theoretical stage model [15,16]. We selected the COM-B (capability, opportunity, motivation and behaviour) model, which synthesises many of the core principles of behaviour change models [16], to develop and analyse our questionnaire. The COM-B model considers behaviour to be an interaction involving three essential components: the capability to perform the behaviour in question, and the opportunity and motivation to carry it out. Research indicates that in order to change behaviour, interventions need to be designed to change one or more of these three components in such a way as to reconfigure behaviour and minimise the risk of relapse.

A 43-item web-based questionnaire was developed following a systematic review of the literature and a two-round Delphi consensus process with members of the PAG. The questionnaire was pilot tested in the participating EU/EEA countries, and validated after translation into the 24 EU official languages, Icelandic and Norwegian. Further questionnaire details are available in Supplement 1 EU EEA antibiotic survey questionnaire with answer options, and Supplement 2 mapping of survey questions to COM-B.

The questionnaire included multiple choice questions, statements testing knowledge using a true or false answer, and statements assessing attitudes and behaviours by seeking agreement using a 5-point Likert scale – strongly agree, agree, neither agree nor disagree, disagree, strongly disagree. In addition, there was an option of ‘I do not understand the question/not applicable’. Further details are available in Supplement 2 Mapping of survey questions to COM-B. The questionnaire consisted of ten sections (Box).

Box **Aspects covered in the online questionnaire, study on healthcare workers’ knowledge, attitudes and behaviours with respect to antibiotics, antibiotic use and antibiotic resistance across 30 EU/EEA countries in 2019**

**Demographic questions**: for each respondent to fill in at the beginning of the survey.
**Capability**: perceived and actual knowledge on human, environmental and animal health factors.
**Opportunity**: level of access to guidelines for managing infections, access to materials to give advice on prudent use of antibiotics and AMR, and questions determining how often they gave out resources and advice.
**Motivation**: level of agreement/disagreement with personal role in helping to control antibiotic resistance, and the connection between their prescribing OR dispensing OR administering of antibiotics and emergence and spread of antibiotic resistant bacteria.
**One health**: level of agreement or disagreement with statements on whether specific environmental and animal health factors contribute to antibiotic resistance.
**Hand hygiene**: self-assessment on being able to list the WHO’s five moments of hand hygiene and whether they needed to perform hand hygiene (as often as recommended) if wearing gloves.
**Information available on antibiotic use and antibiotic resistance or managing infections**: recollection of receiving information about avoiding unnecessary prescribing OR administering OR dispensing of antibiotics, and source(s) of information that had most influenced their views on antibiotic use and resistance.
**Campaign (EAAD evaluation) and training**: level of awareness of EAAD and World Antibiotic Awareness Week (WAAW), and the perceived effectiveness of these campaigns in raising awareness about prudent use of antibiotics and antibiotic resistance within the respondent’s country.
**Future contact**: to determine whether respondents wanted the project team to contact them about their survey responses or other relevant AMR activities, and a question on how they found out about the survey.
**Question for prescribers**: level of confidence in making antibiotic prescribing decisions, access to antibiotic guidelines and confidence in the antibiotic guidelines available to them, individual role in controlling antibiotic resistance, and how often they prescribed antibiotics in the previous week when they would have preferred not to.

AMR: antimicrobial resistance; EAAD: European Antibiotic Awareness Day; EU/EEA: European Union/European Economic Area; WHO: World Health Organization.

A quota sampling approach was used to determine the minimum survey sample size required with the aim of generating a representative sample from different healthcare worker groups in the participating countries. The overall sample size for the study and the sample size per country was determined by calculating 0.2% of all practicing physicians, dentists, pharmacists and 0.1% of all nursing professionals, registered in healthcare personnel statistics for each country individually, and combined from the EU/EEA [17]. The proportion was selected by the project team in collaboration with ECDC to ensure a sufficient but manageable quota sample size/target for in each country. The term physician is defined by the European statistical office (Eurostat) [17] as including generalist medical practitioners (general practitioners (GPs) and other generalist medical practitioners) and specialist medical practitioners (medical specialists and surgical specialists). For the purposes of this survey we chose to use the term medical doctor, and defined it in the questionnaire list as including: general practice, surgeon, specialists - public health, microbiologist and infectious disease physician.

The validated online questionnaire was distributed by the PAG members to healthcare workers in their country, and promoted via social media using #ECDCAntibioticSurvey. Participation was voluntary, and the online questionnaire was open for responses over a 6-week period between 28 January and 4 March 2019. All healthcare workers in each of the 30 participating EU/EEA countries were eligible to complete the online questionnaire.

Data were collected anonymously. All data were held securely in Public Health England’s internal networks and in line with the General Data Protection Regulation 2016/679.

Descriptive statistics for frequency distribution and percentages were used to analyse the respondents’ knowledge and understanding. Comparisons were made using the chi-squared test, and associations were assessed using the odds ratio. A five-point Likert scale was used for several questions and the ‘agreed’ and ‘strongly agreed’ responses were merged and reported as ‘agreed’.

Data were analysed using MS Excel (2010) and STATA statistical software release 15 (StataCorp, College Station, United States (US)). Level of significance was set to p < 0.05.

### Ethical statement

All respondents participated strictly in their professional capacity, and were provided with informed consent prior to participation, according to the Declaration of Helsinki.

Further details of the cross-sectional survey using the CHERRIES checklist for web-based studies is available in Supplement 3 Cherries checklist for ECDC antibiotic survey [18].

## Results

The estimated required quota sample size was 11,931 respondents. In total, 18,365 healthcare workers from the 30 EU/EEA countries responded to the survey, thus exceeding the required quota. Overall demographic data including respondents’ age, gender, years in current profession and professional setting they work more than 50% of the time for the 30 EU/EEA countries are presented in Table 1. Ninety-seven percent of respondents were over the age of 25 years and 70% were women. The respondents predominantly practised in hospitals (49%), the community (22%), or in pharmacies (10%) (Table 1). The number of responses per country and profession, the minimum required quota sample size and additional results are presented in Supplement 4 additional results EU EEA antibiotic survey Tables 1–10 and Figures 1–9.

**Table 1:** Respondents’ age, gender, years in current profession and professional setting where they work more than 50% of the time, EU/EEA, 2019 (n=18,365)

**Age (years)**	**Number of respondents**	
	**n**	**%**
< 18	8	0.0
18–25	556	3.0
26–35	4,307	23.5
36–45	4,325	23.6
46–55	4,695	25.6
56–65	3,716	20.2
> 65	705	3.8
Prefer not to say	53	0.3
**Gender identified with**	**Number of respondents**	
	**n**	**%**
Female	12,850	70.0
Male	5,162	28.1
Prefer not to say	353	1.9
**Years in profession**	**Number of respondents**	
	**n**	**%**
0-2	1,847	10.1
3-5	2,256	12.3
6-10	2,577	14.0
11-15	2,123	11.6
16-20	2,269	12.4
21-25	1,853	10.1
>25	5 440	29.6
**Predominant practice setting**	**Number of respondents**		
	**n**	**%**	
Hospital	8,972	48.9	
Community	3,982	21.7	
Pharmacy	1,742	9.5	
Long-term care facility	1,071	5.8	
Public health institute	664	3.6	
Unknown^a^	583	3.2	
University (as an academic) or research institute	359	2.0	
Governmental organisation	331	1.8	
Professional body	246	1.3	
Industry	233	1.3	
Other^b^	118	0.6	
Not specified	64	0.3	
**Role involves:**	**Number of respondents**		
	**n**	**%**	
Interacting with patients or members of the public in one or more of the following capacities: diagnosing, prescribing, clinical checking of prescriptions, dispensing, administrating, or providing advice on antibiotics	15,059	82	
Contributing to, or leading on antimicrobial stewardship-related programmes, or directly tackling antibiotic resistance in their current role.	5,160	28	

EU/EEA: European Union/European Economic Area.

^a^ Unknown were those who selected ‘other please specify’ but we could not interpret what was written.

^b^ Other was used for those who specified e.g. administrators, managers (not specified of what/where), post-graduate students, retired (not specified which profession), educators of health degrees (where we could not interpret profession), veterinary professionals.

Twenty-four of 30 countries achieved or exceeded the required quota sample size of respondents, two countries achieved more than 70% of their quota sample size of respondents (77% and 84%), while four countries achieved less than 60% of the required quota sample size. The number of responses from medical doctors (including specialists and surgeons), dentists and pharmacists substantially exceeded the required quota sample size for these professions (Supplement 4 Table 1). The number of responses from the nursing profession was only slightly higher (4,772) than the required quota sample size (4,599), and only 55% of the required quota sample size for other healthcare workers was achieved.

Eighty-two percent of respondents (15,059/18,365) stated that their role involved interacting with patients or members of the public in one or more of the following capacities: diagnosing, prescribing, clinical checking of prescriptions, dispensing, administrating, or providing advice on antibiotics (Table 1). Only 28% of respondents stated that they were either contributing to, or leading on antimicrobial stewardship-related programmes, or directly tackling antibiotic resistance in their current role (Table 1).

Ninety-six percent of respondents agreed with the statement ‘I know what antibiotic resistance is’, and 80% agreed with the statement, ‘I have sufficient knowledge about how to use antibiotics appropriately for my current practice’. Responses varied by healthcare worker group (range 44 – 87%) (Table 2) and by country (range 61–93%) (Supplement 4 Table 2).

**Table 2:** Percentage of respondents who agreed with the statements: ‘I know what antibiotic resistance is’ and ‘I have sufficient knowledge about how to use antibiotics appropriately for my current practice’, by professional group, EU/EEA, 2019, (n = 18,365)

Profession	‘I know what antibiotic resistance is’			‘I have sufficient knowledge about how to use antibiotics appropriately for my current practice’		
	Number answering question	Agree or strongly agree		Number answering question	Agree or strongly agree	
		n	%		n	%
Medical doctor	7,351	7,055	96	7,351	6,259	85
Nurse	4,312	4,094	95	4,309	3,340	78
Pharmacist	3,258	3,169	97	3,257	2,758	85
Dentist	1,085	1,029	95	1,085	948	87
Allied health professional	633	585	92	633	277	44
Scientist	461	440	95	461	289	63
Pharmacy Technician	250	239	96	250	192	77
Nursing associate/assistant	250	220	88	250	158	63
Midwife	210	204	97	210	157	75
Other^a^	200	182	91	200	103	52
Other healthcare worker^b^	176	160	91	176	101	57
Unknown^c^	146	126	86	144	86	60
Dental care professional	33	29	88	33	21	64
All professions	18,365	17,532	96	18,359	14,689	80

EU/EEA: European Union/European Economic Area

^a^ Other was used for those who specified e.g. administrators, managers (not specified of what/where), post-graduate students, retired (not specified which profession), educators of health degrees (where we could not interpret profession), veterinary professionals.

^b^ Other healthcare workers was used for those who specified e.g. dispenser, healthcare assistant, homoeopath, hygienist, manager/director (of a health institution), public health promotion specialists, health visitors, pharmacy assistants, nurse coordinator.

^c^ Unknown were those who selected ‘other please specify’ but we could not interpret what was written.

Four of the seven knowledge test statements ‘Antibiotics are effective against viruses’, ‘Antibiotics are effective against cold and flu’, ‘Taking antibiotics has associated side effects or risks such as diarrhoea, colitis, allergies’ and ‘Unnecessary use of antibiotics makes them become ineffective’ were correctly answered by more than 90% of respondents. Two statements ‘Healthy people can carry antibiotic resistant bacteria’ and ‘Antibiotic resistant bacteria can spread from person to person’ were answered correctly by more than 80% of respondents. The statement ‘Every person treated with antibiotics is at an increased risk of antibiotic resistant infection’ was correctly assessed as true by only 75% of respondents, the lowest proportion of the seven questions (Table 3).

**Table 3:** Percentage of respondents who answered each key knowledge question correctly (all healthcare workers), EU/EEA, 2019 (n = 18,354)

Key knowledge question	Correct answer	Correct (%)	Incorrect (%)	Unsure (%)
Antibiotics are effective against viruses	False	97.5	1.7	0.8
Antibiotics are effective against cold and flu	False	97.0	1.7	1.3
Taking antibiotics has associated side effects or risks such as diarrhoea, colitis, allergies	True	96.5	1.9	1.7
Unnecessary use of antibiotics makes them become ineffective	True	94.0	4.1	1.9
Healthy people can carry antibiotic resistant bacteria	True	88.2	3.8	8.0
Antibiotic resistant bacteria can spread from person to person	True	86.9	7.4	5.7
Every person treated with antibiotics is at an increased risk of antibiotic resistant infection	True	75.0	13.7	11.3

EU/EEA: European Union/European Economic Area.

There was a variation in the percentage of respondents answering all seven key knowledge questions correctly across the thirty EU/EEA countries (40%–73%) and professions (29%–58%) (Supplement 4 Tables 3 and 4). No country had 100% of respondents who achieved seven of seven correct answers in the knowledge score, however, most countries (21/30) had more than 50% of respondents answer all the key knowledge questions correctly. Overall, 58% of respondents answered all seven questions correctly, with an average score of 6.35/7 across the 30 EU/EAA countries. Substantial variation was noted between countries (Supplement 4 Table 3). Medical doctors answered all the questions correctly more often than any other respondent category (68%) (chi-squared test=773.8, p < 0.001), followed by pharmacists (59%) (Supplement 4 Table 4).

Findings from the knowledge test questions on environmental and animal health factors and on hand hygiene are provided in Supplement 4 Figures 1 and 2. Only 27% (4,998/18,343) of respondents correctly identified that it is illegal in the EU to use antibiotics to stimulate growth of farm animals; 44% (8,054/18,343) were unsure and 29% (5,291/18,343) believed it was legal practice. Just over half of respondents 56% (9,113/ 16273) stated that they could list the World Health Organization‘s five moments for hand hygiene (Supplement 4 Figure 2).

Seventy-five percent (10,726/14,301) of respondents with direct patient or public involvement agreed that they had easy access to guidelines on managing infections, 68% (9,723/14,299) agreed they had easy access to materials for advising patients about prudent antibiotic use and antibiotic resistance, and 72% (10,293/14,296) agreed that they had good opportunities to provide advice about antibiotic use. Substantial variation was noted by country, profession and setting (Supplement 4 Tables 5-7 and Supplement 4_ Figures 3–5).

Ninety-two percent of respondents (13,908/15,117) with direct patient contact agreed that they knew there was a connection between their prescribing/dispensing/administering of antibiotics and the emergence and spread of antibiotic-resistant bacteria, but only 63% (9,522/15,114) agreed that they have a key role in helping control antibiotic resistance. The proportion of respondents who agreed that they have a key role in helping control antibiotic resistance was higher for those who worked in community settings (65%) than those in hospitals (56%) and other settings (55%).

Sixty-five percent (9,308/14,294) of all respondents had either prescribed, administered or dispensed antibiotics at least once in the week prior to completing the survey. Of these respondents, 17% (2,430/14,294) had given resources (e.g. leaflets or pamphlets), and 55% (7,861/14,294) had provided advice on prudent antibiotic use or management of infections during that week. The most common reasons respondents (n = 13,226) gave for not providing resources or advice as frequently as they prescribed, administered or dispensed antibiotics were because resources were not available (18%), insufficient time (14%), or the patient was disinterested (12%).

Sixty percent of respondents (9,707/16,144) stated that they had received information on avoiding unnecessary prescribing, administering or dispensing of antibiotics in the previous 12 months. Those who had received information received it most frequently at the work place (47%), through published guidelines (45%) or during group training (39%), and felt that the information had contributed to changing their view (58%), or had changed their practice (42%). The majority of those who did not change their practice based on the information received said this was because they were already following the principles of the message (82%), had no control over changing their practice (7%), found the information to be irrelevant to their current practice (5%), or had not had the opportunity (3%). Fifty-five percent of respondents (8,209/14,896) said that they would like to have more information on antibiotic resistance, while 41% (6,254/15,405) stated that prudent antibiotic use and information on antibiotic resistance had been well promoted during national campaigns in their country (Supplement 4_ Figure 6). Fewer than half the respondents across the EU/EEA countries had heard of European Antibiotic Awareness Day (EAAD), (32.4%, 5,028/15,518) or World Antimicrobial Awareness Week (WAAW) (25.6%, 3,942/15,518). Overall, for those who had heard of EAAD and WAAW, the majority were ‘undecided’ (52% and 54%, respectively) on their effectiveness in raising antibiotic awareness in their country, and 27% and 21%, respectively, believed EAAD and WAAW had been effective or very effective in raising antibiotic awareness in their country. Perceived effectiveness of the campaigns in raising awareness was found to vary across countries (Supplementary 4 Figures 7 and 8).Just over one third of respondents (6,791/18,365) stated they were prescribers of antibiotics, of whom 35% prescribed antibiotics daily. Medical doctors (80%) were the largest prescribing group, followed by dentists (12%) nurses (4%) and pharmacists (2%). Most prescribers (90%, 5,870/6,522) agreed that they had a key role in helping control antibiotic resistance, and 90% (5,861/6,520) also said they considered antibiotic resistance when treating a patient. A lower proportion (77%) agreed that they were confident in making antibiotic prescribing decisions, and while most respondents (85%, 5,561/6,519) agreed that they had easy access to the antibiotic guidelines they needed, only 70% (4,520/6,522) said they were confident in the antibiotic guidelines available to them.

Thirty-one percent of prescribers said they would have preferred not to prescribe an antibiotic at least once in the week before completing the survey, but did so anyway. The most common reason for this was fear of patient deterioration or complications, with prescribers reporting that this fear affected their prescribing decision at least once per week (43%), or at least once per day (11%) (Figure). This result varied across countries(Supplement 4 Figure 9). Other reasons for prescribing antibiotics when they would have preferred not to included an uncertain diagnosis (26%), limited time to explain why antibiotics are not indicated (10%), and maintaining the patient relationship (9%). Community prescribers were more likely as hospital prescribers to prescribe antibiotics due to time constraints (27.7% vs 18.2%, p<0.0001) or the need to maintain the patient relationship (22% vs 12.6%, p<0.0001) (Supplement 4_Table 8). More than one third of prescribers (2,085/6,517) disagreed or were undecided as to whether they felt supported to not prescribe unnecessary antibiotics. These proportions varied substantially by country and professional setting (Supplement 4_Tables 9 and 10).

**Figure:**
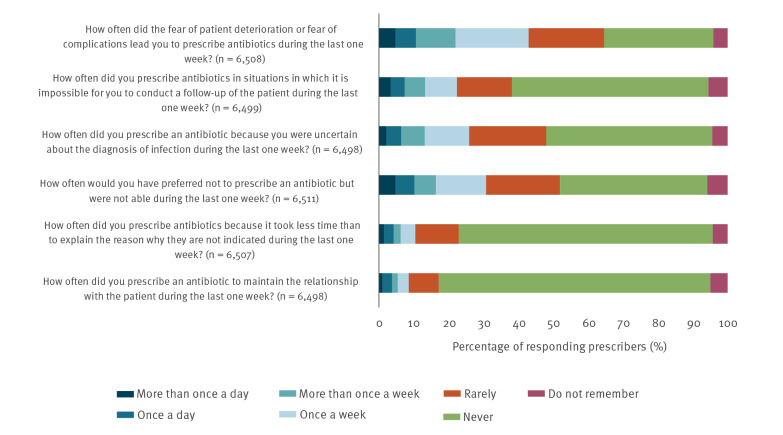
Reasons prescribers initiated antibiotic prescriptions when they would have preferred not to in the previous week, EU/EEA, 2019

EU/EEA: European Union/European Economic Area.

## Discussion

This first EU/EEA-wide survey investigated healthcare workers’ knowledge of antibiotics, antibiotic use and antibiotic resistance, and whether they carry out the recommended behaviours on antibiotic use. Its results show a variation in healthcare workers’ responses across EU/EEA countries, and provide baseline evidence that may be useful for the development and evaluation of future interventions, both in individual countries and more broadly across the EU/EEA.

The study exceeded the calculated required quota sample size, and there was also good representation from all the core healthcare professional groups who prescribe, administer or dispense antibiotics. This indicates that the findings are broadly representative of the relevant healthcare worker categories across the EU/EEA. Two limitations to consider are that those who responded to the survey may have an interest in tackling antimicrobial resistance (AMR) and 40% of respondents were medical doctors. However, it is worth noting that the majority of respondents do not have a formal role in tackling AMR, and all settings were represented. Another limitation was that while the estimated required quota was met/exceeded for the majority of countries, some countries had a higher representation from a particular healthcare professional group than others. There was also a higher response rate from women, although this is unsurprising since by 2016, 15 of the EU countries reported a higher number of female physicians (cf.d with 9 countries which reported a higher number of male physicians). In addition, a substantial proportion of the nursing profession is female [17]. Also, evidence suggests that women may, in general, be more willing to participate in online surveys than men [19].

The findings of the survey highlight the need to continue to raise awareness about prudent use of antibiotics and antibiotic resistance, and also to enhance healthcare workers’ engagement in addressing these issues. They also highlight the need to design interventions based on education, resources and guidelines, which focus specifically on promoting behaviour that leads to prudent prescription, dispensing, and administration of antibiotics.

As found in previous studies (predominantly focused on physicians), knowledge and perceived knowledge about antibiotics, antibiotic use and antibiotic resistance was high among healthcare workers, with more than 80% of respondents acknowledging the connection between prescribing, dispensing and administering antibiotics and the emergence and spread of antibiotic resistance [9-13,20,21]. However, the present study also provides further evidence that while healthcare workers are aware of the potential threat of antibiotic resistance, knowledge is not the only factor that affects healthcare workers’ antibiotic-related behaviours [22-24].

Although more than 80% of respondents across all healthcare worker categories correctly answered the questions on the use of antibiotics, a much lower proportion were able to correctly answer questions related to the development and spread of antibiotic resistance. There was also a wide variation across professions in the proportion of respondents answering all seven knowledge questions correctly. The groups with the lowest knowledge and the groups who perceived they did not have sufficient knowledge on how to use antibiotics appropriately for their current practice should be targeted by future educational campaigns.

It is reassuring that the survey questions relating to key and consistent messages promoted as part of previous EAAD campaigns throughout Europe i.e. that antibiotics are not effective against viruses, colds and flu, had the highest proportion of correct answers, and also that healthcare workers answered these questions more accurately than the general public in the Eurobarometer studies [4-7]. However, other topics were less well understood, such as ‘Every person treated with antibiotics is at an increased risk of antibiotic resistant infection’, ‘Antibiotic resistant bacteria can spread from person to person’, and ‘Healthy people can carry antibiotic resistant bacteria’. These important topic areas should therefore be targeted in future educational interventions.

Increasing engagement and promoting a sense of shared responsibility to tackle antibiotic resistance at individual, population and government levels, are potentially important means of bringing about behaviour change. One method that has previously been used is to focus on setting implementation intentions by pledging to take concrete action. This can be accomplished through, for example, joining a collective movement to cause change. Implementation intention is a method of encouraging individuals to decide in advance when, where and how they will act in order to reach a particular goal or objective. This approach uses what is described as if-then planning: if X happens, then I will do Y. For example, one of the pledges for general (primary care) practitioners used in the United Kingdom’s (UK) led Antibiotic Guardian campaign to support the UK Antimicrobial Resistance strategy states: ‘The next time I intend to prescribe antibiotics for a self-limiting infection to a patient with high expectations of antibiotic treatment, I will use a delayed/backup prescription’.

The use of implementation intentions has been shown through meta-analyses to support both individuals and groups in bridging their intention-behaviour gaps [25,26]. Evaluation of the Antibiotic Guardian campaign has shown that the if-then approach increased commitment to tackling antibiotic resistance in both healthcare workers and members of the public, increased self-reported knowledge and changed self-reported behaviour. This was particularly the case among people with prior awareness of antibiotic resistance [27,28]. Online pledge schemes are one example of how a communication campaign can be an effective yet inexpensive way to engage people with issues around antibiotic resistance, especially those with some prior awareness of the topic [27-29].

Most healthcare workers in our study who had direct patient or public involvement reported that they prescribed, dispensed or administered antibiotics at least once per week. While the vast majority of these interactions did not involve providing any written or oral advice, the most common barriers stated for not providing written resources to patients were that no resources were available, they had insufficient time, or the patient was not interested. It is therefore important that healthcare workers have access to appealing educational resources about antibiotics and antibiotic resistance when prescribing, dispensing or administering antibiotics. Although some materials about the importance of using antibiotics appropriately are available for the general public, these are often concerned with communicating the risks of antibiotic resistance or the need for prudent use of antibiotics in general terms. They are not patient-specific materials which provide information on, for example, the importance of taking antibiotics exactly as prescribed (dose/duration), and not to save them for later. Patient brochures covering topics such as ‘When should I worry?‘ [30], and ‘Treating your infection‘ [31], summarise the likely duration of self-limiting infections and offer advice on when to re-consult with a health professional. These, alongside self-care recommendations are examples of patient resources that could be promoted for use by healthcare workers across EU/EEA countries, adapted as appropriate for local/national context [32-34].

Our findings also point to the importance of ongoing training to enhance communication skills for those with direct patient contact. A cluster randomised control trial including primary care practices in five European countries representing north, south and central Europe (Belgium, the Netherlands, Poland, Spain and the UK), has previously shown that Internet-based training to enhance the communication skills of prescribers, including the use of a patient information booklet, achieved reductions in antibiotic prescription for respiratory tract infections across language and cultural boundaries [21]. As this trial took place within the context of a research project, national authorities would need to commit specific funds for such an approach to be sustainable outside a research setting.

Regarding the motivation for prescribing antibiotics, the findings highlight that clinicians‘ fear of serious bacterial infection and attempts to safeguard against the deterioration of a patient‘s health are important factors in their inappropriate prescribing of antibiotics. This points to the importance of developing rapid diagnostic tests/point-of-care tests that would remove this uncertainty, and thereby allow for more appropriate prescribing. Parallel media campaigns, which inform the public that they should trust their healthcare professional if they decide that antibiotics are not necessary for them could also be developed. Whatever interventions are implemented, it is important to evaluate their effectiveness, thereby ensuring a process of continual improvement. Countries could use the data from this study as a baseline for such evaluations, and use the survey tool as a means of assessing changes in the measured variables. However, it is important that the appropriate sample size for each country is determined at a national level.

At EU/EEA level, it may be beneficial to consider developing a data repository platform to which countries can submit the results of their locally deployed survey findings, thereby facilitating benchmarking and monitoring at European level. In addition, this healthcare worker survey could be run at an EU/EEA-wide level at regular intervals or by individual countries in a similar manner to the Eurobarometer survey that focuses on the general public [4-7],

While it is important to continue EU-level awareness campaigns such as the EAAD, interventions at national and local level are key to changing antibiotic-related behaviours of healthcare workers. Previous systematic reviews have shown that the effectiveness of an intervention on antibiotic prescribing depends to a large extent on the particular prescribing behaviour and any barriers to change that may exist within the targeted community. In addition, multi-faceted educational interventions occurring on multiple levels are only effective after addressing such local barriers to change [21,24,35]. Educational training and communication materials for healthcare workers in Europe should take this into account, and behaviour change strategies should be the aim with any intervention.

## Conclusion

While several studies in Europe have assessed antibiotic-related knowledge and attitudes of members of the public, healthcare students or individual professional groups, there is a paucity of equivalent literature that focuses on healthcare workers. To our knowledge, this is the first multi-country and multi-professional study on the knowledge, attitudes and behaviours of healthcare workers regarding antibiotics, antibiotic use and antibiotic resistance, and it has identified important knowledge gaps such as ‘Every person treated with antibiotics is at an increased risk of antibiotic resistant infection’, ‘Antibiotic resistant bacteria can spread from person to person’, and ‘Healthy people can carry antibiotic resistant bacteria’ that need to be addressed. While some of these can be addressed at EU/EEA level, individual countries should review the data presented in this study, and use these to develop a tailored approach for their own context.

## Note

The following contributors from the #ECDCAntibioticSurvey Project Advisory Group were not contactable for the approval of the final version of the article before publication despite several efforts made by the corresponding author over the course of 8 weeks: Barbora Macková (Czech Republic); Flora Kontopidou (Greece); Kate Vulāne (Latvia); Lenneke Schrier (European Academy of Paediatrics); Maria Foteinea (Greece); Duarte Pedro De Sousa Tavares (Portugal); Orsolya Réka Süli (European Medical Students’ Association)**.**

## #ECDCAntibioticSurvey Project Advisory Group

Reinhild Strauss (Austria); Vinciane Charlier, Samuel Coenen (Belgium); Miranda Sertić, Marina Payerl-Pal (Croatia); Linos Hadjihannas, Costas A. Constantinou (Cyprus); Barbora Macková (Czech Republic); Lisa Bugge-Toft (Denmark); Pille Märtin, Mailis Hansen (Estonia); Outi Lyytikäinen, Jari Jalava (Finland); Anne Berger-Carbonne, Mélanie Colomb-Cotinat (France); Flora Kontopidou, Maria Foteinea (Greece); Martin Cormican, Audrey Lambourn (Ireland); Francesca Furiozzi, Michela Sabbatucci (Italy); Elīna Dimiņa, Kate Vulāne (Latvia); Virginija Kanapeckienė, Jolanta Kuklytė (Lithuania); Peter Zarb, Michael A. Borg (Malta); Renske Eilers (the Netherlands); Harald Pors Muniz (Norway); Waleria Hryniewicz, Beata Mazińska (Poland); Duarte Pedro De Sousa Tavares (Portugal); Livia Cioran, Alexandra Cucu (Romania); Eva Schreterova (Slovakia); Mitja Vrdelja, Maja Subelj (Slovenia); Rocío Bueno Parralo, Antonio López Navas (Spain); Agneta Andersson, Karin Carlin (Sweden); Jacqui Reilly, Diane Ashiru-Oredope (UK); Lea Pfefferle, Task Force on Antibiotics in Dentistry (Council of European Dentists); Petr Horák, Steffen Amann (European Association of Hospital Pharmacists); Andreas Trobisch, Lenneke Schrier (European Academy of Paediatrics); Tanguy Pinedo-Tora, Alyette Greiveldinger (European Dental Students‘ Association); Elena Carrara, Nico T. Mutters (European Committee on Infection Control); Charles Price (European Commission); Mathias Maucher (European Federation of Public Service Unions); Michele Calabrò (European Health Management Association); Pascal Garel, Laurie Andrieu (European Hospital and Healthcare Federation); Laura Alonso Irujo, María Santacreu García (European Joint Action on Antimicrobial Resistance and Healthcare-Associated Infections, EU-JAMRAI); Kitty Mohan, Sara Launio (European Junior Doctors Association); Orsolya Réka Süli (European Medical Students‘ Association); Ivana Silva (European Medicines Agency); Mervi Jokinen (European Midwives Association); Marta Simões, Ruben Viegas (European Pharmaceutical Students‘ Association); Ann Marie Borg, Sascha Marschang (European Public Health Alliance); Céline Pulcini (European Society of Clinical Microbiology and Infectious Diseases Study Group for Antimicrobial stewardship); Ber Oomen, Jeannette Verkerk (European Specialist Nurses Organisations); Ilaria Giannico, Paul Garassus (European Union of Private Hospitals); Roberto Bertollini, Melina Raso (Health First Europe); Tímea Rezi-Kató (MedTech Europe); Jan De Belie, Ilaria Passarani (Pharmaceutical Group of the European Union); Jacques de Haller, Carole Rouaud (Standing Committee of European Doctors); Jo Bosanquet, Wendy Nicholson (WHO Collaborating Centre for Nurses); Cristiana Salvi (WHO/Europe).

## Supplementary Data

364000 Supplementary Material 1

## Supplementary Data

566000 Supplementary Material 2

## Supplementary Data

1175000 Supplementary Material 3

## Supplementary Data

424000 Supplementary Material 4
