# Supplementary material for: Healthcare workers’ knowledge, attitudes and behaviours with respect to antibiotics, antibiotic use and antibiotic resistance across 30 EU/EEA countries in 2019
Source: Euro Surveill. 2021 Mar 25;26(12):1900633. doi: 10.2807/1560-7917.ES.2021.26.12.1900633 (PMC7995558; doi:10.2807/1560-7917.ES.2021.26.12.1900633)
Supplement: Supplementary Material 1 [file 1900633_Supplementary_material_1.pdf]

This supplementary material is hosted by Eurosurveillance as supporting information alongside the article 'Healthcare workers' knowledge, attitudes and behaviours with respect to antibiotics, antibiotic use and antibiotic resistance across 30 EU/EEA countries in 2019', on behalf of the authors, who remain responsible for the accuracy and appropriateness of the content. The same standards for ethics, copyright, attributions and permissions as for the article apply. Supplements are not edited by Eurosurveillance and the journal is not responsible for the maintenance of any links or email addresses provided therein.

Page 1

Survey of healthcare workers knowledge and attitudes about antibiotics and antibiotic resistance

Dear Respondent,

You are invited to complete the following survey of healthcare workers about their knowledge and attitudes about antibiotics and antibiotic resistance, funded by the European Centre for Disease Prevention and Control (ECDC).

Previous studies have mostly focused on the general public and medical students, highlighting a gap in the understanding of these topics by healthcare workers and by other health students.

ECDC and Public Health England (who have been commissioned to develop and implement the survey) are seeking responses from healthcare workers, including doctors, nurses, pharmacists and hospital managers as well as clinical scientists, physiotherapists, nursing assistants, dental/pharmacy technicians, public health teams and health students.

We would really value you completing the survey by 14 February 2019.

We believe the survey will take 5 to 10 minutes to complete. It includes predominantly multiple choice questions. The red asterisks (*) are used for questions that require a mandatory answer before proceeding to the next page of the survey.

Please feel free to cascade the link of the survey widely to colleagues (and health students).

If you have any questions about this survey, or difficulty in completing the survey, please contact Dr Diane Ashiru-Oredope (project lead) via espaur@phe.gov.uk.

Are you involved in diagnosis, prescribing, clinical checking prescriptions, dispensing, administration, or provision of advice of antibiotics to patients or members of the public? *

For students, will you be involved in diagnosis, prescribing, dispensing, administration, or provision of advice of antibiotics to patients or members of the public in the near future?

- Yes
- No

About you

In what country do you currently practice? If you are an undergraduate student - in what country are you currently studying?*

This survey is for EU/EEA countries. If you are from a non-EU country, please select 'other' and there will be a follow-up question requesting you put the country you work/study in.

[Austria ▼] *Options: The 30 EU/EEA countries of focus for the study along with "Other".*

Please specify which continent.*

[-- Please Select -- ▼] *Options: The 7 continents.*

Please specify in which country you practice.*

[ ]

What is your core profession?

Note: subsequent question available on speciality

*

- ◯ Medical doctor (e.g. general practice, surgeon, specialists - public health, microbiologist, infectious disease physician)
- ◯ Nurse (e.g. general practice, surgeon, specialists - public health, infection prevention/control)
- ◯ Nursing associate / Assistant nurse
- ◯ Midwife
- ◯ Dentist
- ◯ Dental care professional (e.g. dental nurses, dental technicians)
- ◯ Pharmacist
- ◯ Pharmacy technician
- ◯ Allied Health Professional (e.g. physiotherapist, paramedic, podiatrist, radiographer)
- ◯ Scientist (e.g. microbiologist, biomedical scientist)
- ◯ Undergraduate health student (e.g. medical, dental, nursing, pharmacy, health sciences)
- ◯ Other, please specify

[ ]

What is your predominant role? (i.e.>50% of your time)*

- ◯ Generalist (e.g. general medicine/nursing, community pharmacy, family doctors)
- ◯ Specialist non infection (e.g. intensive care, surgical, cardiology, specialist pharmacy, paediatrics)
- ◯ Specialist infection (e.g.clinical microbiology, infectious diseases, infection control, micro pharmacy)
- ◯ Management (e.g hospital manager, CEO, commissioner)
- ◯ Public Health (e.g. working in institutes/departments - local, regional or national)
- ◯ Academia/Research
- ◯ Scientist (e.g. microbiologist, biomedical scientist)
- ◯ Undergraduate health student (e.g. medical, dental, nursing, pharmacy, health sciences)
- ◯ Other, please specify

[ ]

Where do you predominantly practice? (i.e. >50% of your time)*

- ◯ Hospital (any hospital type)
- ◯ Long-term Care Facility/Service
- ◯ Community
- ◯ Industry
- ◯ Professional body
- ◯ Government Organisation
- ◯ Public Health institute
- ◯ University (as an Academic) or research institute
- ◯ Pharmacy
- ◯ Other, please specify

How many years have you been practicing in your current profession?*

-- Please Select --

What is your age?*

-- Please Select --

What gender do you most identify with?*

- [ ] Male
- [ ] Female
- [ ] I prefer not to say

How many years have you been practicing in your current profession?
Options:
0-2 years
3-5 years
6-10 years
11-15 years
16-20 years
21-25 years
>25 years
Not yet qualified (students)

What is your age?
Options:
<18 years
18-25 years
26-35 years
36-45 years
46-55 years
56-65 years
>66 years
I prefer not to say

Which of the following social media networks do you mainly use for professional activities?*

Select no more than 2.

- [ ] Twitter
- [ ] Facebook
- [ ] LinkedIn
- [ ] Google+
- [ ] YouTube
- [ ] Instagram
- [ ] I do not use social media
- [ ] Other, please specify

In your current role are you contributing to/leading antimicrobial stewardship programmes or tackling AMR?*

e.g. member of organisation focused on tackling AMR or an infection related role

- ( ) Yes
- ( ) No
- ( ) I don't understand this question

Antibiotic use and antibiotic resistance

To what extent do you agree or disagree with the following statements?

1=Strongly Disagree; 2=Disagree; 3=Undecided; 4=Agree; 5=Strongly Agree
IDU=I do not understand the question; N/A=Not applicable
*

Individual = Patient or member of the public

	1	2	3	4	5	IDU	N/A
I know what antibiotic resistance is	○	○	○	○	○	○	○
I know there is a connection between my prescribing OR dispensing OR administering of antibiotics and emergence and spread of antibiotic resistant bacteria	○	○	○	○	○	○	○
I know what information to give to individuals about prudent use of antibiotics and antibiotic resistance	○	○	○	○	○	○	○
I have sufficient knowledge about how to use antibiotics appropriately for my current practice	○	○	○	○	○	○	○
I have a key role in helping control antibiotic resistance	○	○	○	○	○	○	○

Please answer whether you believe these statements are true or false.
*

	True	False	Unsure
Antibiotics are effective against viruses	○	○	○
Antibiotics are effective against cold and flu	○	○	○
Unnecessary use of antibiotics make them become ineffective	○	○	○
Taking antibiotics has associated side effects or risks such as diarrhoea, colitis, allergies	○	○	○
Every person treated with antibiotics is at an increased risk of antibiotic resistant infection	○	○	○
Antibiotic resistant bacteria can spread from person to person	○	○	○
Healthy people can carry antibiotic resistant bacteria	○	○	○
The use of antibiotics to stimulate growth in farm animals is legal in the EU	○	○	○

Antibiotic use and antibiotic resistance

To what extent do you agree or disagree with the following statements?

1=Strongly Disagree; 2=Disagree; 3=Undecided; 4=Agree; 5=Strongly Agree
IDU=I do not understand the question; N/A=Not applicable

Individiual = Patient or member of the public*

	1	2	3	4	5	IDU	N/A
I have easy access to guidelines I need on managing infections	○	○	○	○	○	○	○
I have easy access to the materials I need to give advice on prudent antibiotic use and antibiotic resistance	○	○	○	○	○	○	○
I have good opportunities to provide advice on prudent antibiotic use to individuals	○	○	○	○	○	○	○

Considering the **last one week** only in your **clinical practice**, please rate how frequently the statements apply to you. If a question is not applicable then please choose N/A.

Note: This section includes separate questions about providing **advice** and **resources to individuals**

Individual = patient or member of the public

1=Once a day; 2=More than once a day; 3=Once a week; 4=More than once a week; 5=rarely; 6=never;
IDR=I do not remember; N/A=Not Applicable

*

	1	2	3	4	5	6	IDR	N/A
How often did you prescribe OR dispense OR administer antibiotics during the last one week?	○	○	○	○	○	○	○	○
How often did you give out resources (e.g. leaflets or pamphlets) on prudent antibiotic use or management of infections to individuals during the last one week?	○	○	○	○	○	○	○	○
How often did you give out advice related to prudent antibiotic use or management of infections to an individual during the last one week?	○	○	○	○	○	○	○	○

If you were not able to give out advice or resources as frequently as you prescribed

OR dispensed OR administered antibiotics, why was this?

Select all that apply

- [ ] Patient does not require information
- [ ] Patient uninterested in information
- [ ] Insufficient time
- [ ] Difficulty getting patient to understand diagnosis
- [ ] Language barriers
- [ ] No resources available
- [ ] I was not sure what advice to provide
- [ ] I was able to give out advice or resources as needed
- [ ] Not applicable
- [ ] Other, please specify

Antibiotic use and antibiotic resistance

To what extent do you agree or disagree that the following environmental and animal health factors are important in contributing to antibiotic resistance in bacteria from humans?

1=Strongly Disagree; 2=Disagree; 3=Undecided; 4=Agree; 5=Strongly Agree
IDU=I do not understand the question
*

	1	2	3	4	5	IDU
Environmental factors such as waste water in the environment	○	○	○	○	○	○
Excessive use of antibiotics in livestock and food production	○	○	○	○	○	○

In the management of infections, which of these do you use regularly?

Select no more than 3.

- ☐ Clinical practice guidelines
- ☐ Documentation from the pharmaceutical industry
- ☐ Medical representatives from industry
- ☐ Previous clinical experience
- ☐ Continuing education training courses
- ☐ Infection specialists
- ☐ Scientific journals
- ☐ Professional resources/publications
- ☐ Social media
- ☐ None of the above
- ☐ I do not know
- ☐ Other, please specify

Please state "Yes", "No" or "Unsure" in regards to your knowledge on the following statements regarding **hand hygiene.** *

	Yes	No	Unsure
I can list the WHO’s five moments of hand hygiene	○	○	○
I need to perform hand hygiene (as often as recommended) if I have used gloves in contact with patients or biological material	○	○	○

Information available on antibiotic use and antibiotic resistance

In the **last 12 months**, do you remember receiving any information about avoiding unnecessary prescribing OR administering OR dispensing of antibiotics?*

- Yes
- No
- Unsure

If yes, how did you first get this information about avoiding unnecessary prescribing OR administering OR dispensing of antibiotics?*

Select all that apply.

- Colleague or peer
- My workplace
- Media (TV/Radio) adverts
- Social Media
- Newspaper
- Published guidelines
- Training - conference/group
- Training - one to one detailing
- Government policy
- Scientific organisation
- My professional body (e.g. medical/pharmaceutical/nursing societies)
- Audit and feedback
- Other, please specify

Did the information contribute to **changing your views** about avoiding unnecessary prescribing OR administering OR dispensing of antibiotics?*

- Yes
- No
- Unsure

Which source(s) of information has had the **most influence** on changing your views?*

Select no more than 2.

- Colleague or peer
- My workplace
- Media (TV/radio) adverts
- Social media
- Newspaper
- Published guidelines
- Training - conference/group
- Training - one to one detailing
- Government policy
- Scientific organisation
- My professional body (e.g. medical/pharmaceutical/nursing societies)
- Audit and feedback
- Other, please specify

On the basis of the information you received, **have you changed your practice** on prescribing OR administering OR dispensing of antibiotics?*

- Yes
- No
- Unsure

If yes, please list what has had the **most** influence on changing your practice?*

If no, why not?*

Please tick one

- I was already following the principles of the message(s)
- I have not had the opportunity
- I forgot about the message
- I do not think the message is important
- I have no control over it
- The information was not relevant for my practice
- Other, please specify

Campaign and Training questions

At what level do you think it is most effective to tackle resistance to antibiotics?*

Select no more than 2.

- ☐ Individual level (public)
- ☐ Individual level (prescribers)
- ☐ Individual level (all healthcare workers)
- ☐ Environmental/Animal Health
- ☐ Regional/National Level
- ☐ EU/Global
- ☐ Action at all levels needed
- ☐ I do not know

What initiatives are you aware of in your country which focus on antibiotic awareness and resistance? *

Select all that apply

- ☐ TV or Radio advertising for the public
- ☐ Toolkits and resources for healthcare workers
- ☐ National or regional guidelines on management of infections
- ☐ Awareness raising from professional organisations
- ☐ Conference/Events focused on tackling antibiotic resistance
- ☐ National or regional posters or leaflets on antibiotic awareness
- ☐ Newspaper (national) articles on antibiotic resistance
- ☐ National campaign
- ☐ World Antibiotic Awareness Week/European Antibiotic Awareness Day
- ☐ I am not aware of any initiatives
- ☐ Other, please specify

To what extent do you agree or disagree with the following statements regarding the national initiatives about prudent use of antibiotics in your country?

1=Strongly Disagree; 2=Disagree; 3=Undecided; 4=Agree; 5=Strongly Agree
IDR=I do not remember; N/A=Not applicable

*

If you are not aware of any national initiatives in your country, please choose "N/A"

	1	2	3	4	5	IDR	N/A
There has been good promotion of prudent use of antibiotics and antibiotic resistance in my country	○	○	○	○	○	○	○
I believe the national campaign has been effective in reducing unnecessary antibiotic use and controlling antibiotic resistance	○	○	○	○	○	○	○

Does your country have a national action plan on antimicrobial resistance?*

- Yes
- No
- Unsure

Have you heard of European Antibiotic Awareness Day (EAAD) or World Antibiotic Awareness Week (WAAW)?*

	Yes	No	Unsure
EAAD			
WAAW			

How effective do you believe EAAD and WAAW have been in raising awareness about prudent use of antibiotics and antibiotic resistance in your country?

1=Very Ineffective; 2=Ineffective; 3=Undecided; 4=Effective; 5=Very Effective
IDU=I don't understand the question
*

	1	2	3	4	5	IDU
EAAD						
WAAW						

On which topics would you like to receive more information?*

- Resistance to antibiotics
- How to use antibiotics
- Medical conditions for which antibiotics are used
- Prescription of antibiotics
- Links between the health of humans, animals and the environment
- None
- Other, please specify

How did you find out about the survey? *

- National organisation
- Government organisation
- Local organisation (e.g. your hospital)
- Social media
- Professional body/organisations
- Conference/event
- Colleague
- University/academia
- Other, please specify

May we contact you in the future about:*

	Yes	No
Your survey responses		
Other relevant AMR activities		

Please provide your name

Please provide your email address

Do you currently prescribe antibiotics or are you currently an undergraduate health student?*

-- Please Select --

Options:
Yes, I am a Prescriber
Yes, I am a Student
Neither

Page 9

Prescriber questions - only for those who state that they are prescribers

How often do you prescribe antibiotics?*

-- Please Select -- ▼

Options:
Everyday
Weekly
Monthly
Quarterly
Yearly

To what extent do you agree or disagree with the following statements?

1=Strongly Disagree; 2=Disagree; 3=Undecided; 4=Agree; 5=Strongly Agree;
IDU=I do not understand the question

*

	1	2	3	4	5	IDU
I am confident making antibiotic prescribing decisions	○	○	○	○	○	○
I have confidence in the antibiotic guidelines available to me	○	○	○	○	○	○
I have a key role in helping control antibiotic resistance	○	○	○	○	○	○
I consider antibiotic resistance when treating a patient	○	○	○	○	○	○
I have easy access to antibiotic guidelines I need to treat infections	○	○	○	○	○	○
I feel supported to not prescribe antibiotics when they are not necessary	○	○	○	○	○	○

Considering the **last one week only:**

1=Once a day; 2=More than once a day; 3=Once a week; 4=More than once a week; 5=rarely; 6=never;
IDR=I do not remember

*

	1	2	3	4	5	6	IDR
How often would you have preferred not to prescribe an antibiotic but were not able during the last one week?	○	○	○	○	○	○	○
How often did the fear of patient deterioration or fear of complications lead you to prescribe antibiotics during the last one week?	○	○	○	○	○	○	○
How often did you prescribe antibiotics because it took less time than to explain the reason why they are not indicated	○	○	○	○	○	○	○

during the last one week?

How often did you stop an antibiotic prescription earlier than the prescribed course length during the last one week?

How often did you prescribe antibiotics in situations in which it is impossible for you to conduct a follow-up of the patient during the last one week?

How often did you prescribe an antibiotic to maintain the relationship with the patient during the last one week?

How often did you prescribe an antibiotic because you were uncertain about the diagnosis of infection during the last one week?

How often did you prescribe a shorter course of treatment as compared to available guidelines during the last one week?

How often did you discontinue early (within three days after initiation) a treatment because bacterial infection was not likely after all during the last one week?

What strategies do you employ to prescribe antibiotics prudently?

*

Select all that apply.

- [ ] Delayed prescribing/ back-up prescribing (delayed prescribing is a method whereby a prescription is issued by a health professional for use by the patient at a later date, if their symptoms do not improve)
- [ ] Patient education
- [ ] New patient consultation
- [ ] None
- [ ] Other, please specify

Undergraduate health student questions - only for those who state that they are students/trainees

This section is for undergraduate health students. Residents, interns, junior doctors should complete prescriber questions.

What career are you currently studying for?

*

- Medicine
- Nursing
- Pharmacy
- Dentistry
- Scientist
- Other, please specify

How many years is the undergraduate degree for your chosen career?

*

- 3 years
- 4 years
- 5 years
- > 5 years
- Other, please specify

What year of your studies are you in?

*

- 1st year
- 2nd year
- 3rd year
- 4th year
- 5th year or later
- Other, please specify

What university are you studying at?*

Have you had any teaching about antibiotic treatment and prudent antibiotic use during your undergraduate degree?*

	Yes	No	Unsure
Prudent antibiotic use			
Management of infections (diagnosis and antibiotic treatment)			

Have any of your examinations included questions about antibiotic treatment or prudent use of antibiotics? *

	Yes	No	Unsure
Prudent antibiotic use			
Management of			

infections (diagnosis and antibiotic treatment)

Which of the following methods of teaching have been used to teach you about prudent use of antibiotics/antibiotic treatment and how useful would you rate them?

1=Not very useful; 2=Not useful; 3=Undecided; 4=Useful; 5=Very useful
IDU=I do not understand the question; N/A=Not applicable
*

If the teaching method was not used, please choose "N/A".

	1	2	3	4	5	IDU	N/A
Lectures (with >15 people)	○	○	○	○	○	○	○
Small group teaching (with <15 people)	○	○	○	○	○	○	○
Discussions of clinical cases and vignettes	○	○	○	○	○	○	○
Active learning assignments (e.g. article reading, group work, preparing an oral presentation)	○	○	○	○	○	○	○
E-learning	○	○	○	○	○	○	○
Role play or communication skills sessions dealing with patients demanding antibiotic training	○	○	○	○	○	○	○
Infectious diseases clinical placement (i.e. clinical rotation or training in infectious diseases, involving patients)	○	○	○	○	○	○	○
Microbiology clinical placement	○	○	○	○	○	○	○
Peer or near peer-teaching (i.e.teaching led by other students or recently qualified doctors)	○	○	○	○	○	○	○

Have you had any practical experience of your future profession? (e.g. internship or placement)*

○ Yes
○ No
○ I do not understand the question.

How do you think teaching on antibiotic treatment and/or prudent use of antibiotics can be improved?
