# Supplementary material for: Healthcare workers’ knowledge, attitudes and behaviours with respect to antibiotics, antibiotic use and antibiotic resistance across 30 EU/EEA countries in 2019
Source: Euro Surveill. 2021 Mar 25;26(12):1900633. doi: 10.2807/1560-7917.ES.2021.26.12.1900633 (PMC7995558; doi:10.2807/1560-7917.ES.2021.26.12.1900633)
Supplement: Supplementary Material 2 [file 1900633_Supplementary_material_2.pdf]

This supplementary material is hosted by *Eurosurveillance* as supporting information alongside the article 'Healthcare workers' knowledge, attitudes and behaviours with respect to antibiotics, antibiotic use and antibiotic resistance across 30 EU/EEA countries in 2019', on behalf of the authors, who remain responsible for the accuracy and appropriateness of the content. The same standards for ethics, copyright, attributions and permissions as for the article apply. Supplements are not edited by *Eurosurveillance* and the journal is not responsible for the maintenance of any links or email addresses provided therein

Supplement 2: Mapping of survey questions to COM-B

Healthcare workers' knowledge, attitudes and behaviours with respect to antibiotics, antibiotic use and resistance in 30 EU/EEA countries

All statements seeking agreement were a 5-point Likert scale – strongly agree, agree, neither agree nor disagree, disagree, strongly disagree. In addition, there was an option of I do not understand the question/not applicable

Section 1 - Capability - perceived and actual knowledge

To what extent do you agree or disagree with the following statements? Individual = patient or member of the public

- I know what antibiotic resistance is
- I know what information to give to individuals about prudent use of antibiotics and antibiotic resistance
- I have sufficient knowledge about how to use antibiotics appropriately for my current practice

Please answer whether you believe these statements are true or false.

1. Antibiotics are effective against viruses
2. Antibiotics are effective against cold and flu
3. Unnecessary use of antibiotics makes them become ineffective
4. Taking antibiotics has associated side effects or risks such as diarrhoea, colitis, allergy
5. Every person treated with antibiotics is at an increased risk of antibiotic resistant infection
6. Antibiotic resistant bacteria can spread from person to person
7. Healthy people can carry antibiotic resistant bacteria

Section 2 - Opportunity

To what extent do you agree or disagree with the following statements? Individual = patient or member of the public

- I have easy access to guidelines I need on managing infections
- I have easy access to the materials I need to give advice on prudent antibiotic use and antibiotic resistance
- I have good opportunities to provide advice on prudent antibiotic use to individuals

Considering the last one week only in your clinical practice, please rate how frequently the statements apply to you. If a question is not applicable then please choose N/A.

- How often did you prescribe OR dispense OR administer antibiotics during the last one week?
- How often did you give out resources (e.g. leaflets or pamphlets) on prudent antibiotic use or management of infections to individuals during the last one week?
- How often did you give out advice related to prudent antibiotic use or management of infections to an individual during the last one week?

If you were not able to give out advice or resources as frequently as you prescribed OR dispensed OR administered antibiotics, why was this?

Section 3 - Motivation

To what extent do you agree or disagree with the following statements?

- I know there is a connection between my prescribing OR dispensing OR administering of antibiotics and emergence and spread of antibiotic resistant bacteria
- I have a key role in helping control antibiotic resistance

Section 4 - One health

To what extent do you agree or disagree that the following environmental and animal health factors are important in contributing to antibiotic resistance in bacteria from humans?

- Environmental factors such as waste water in the environment
- Excessive use of antibiotics in livestock and food production

Please answer whether you believe these statements are true or false.

- The use of antibiotics to stimulate growth in farm animals is legal in the EU

Section 5 - Hand hygiene

Please state "Yes", "No" or "Unsure" in regards to your knowledge on the following statements regarding hand hygiene.

- I can list the WHO's five moments of hand hygiene
- I need to perform hand hygiene (as often as recommended) if I have used gloves in contact with patients or biological material

Section 6 - Information available on antibiotic use and antibiotic resistance or managing infections

- In the management of infections, which of these do you use regularly? Select no more than 3.
    - Clinical practice guidelines
    - Documentation from the pharmaceutical industry
    - Medical representatives from industry
    - Previous clinical experience
    - Continuing education training courses
    - Infection specialists
    - Scientific journals
    - Professional resources/publications
    - Social media
    - None of the above

- In the last 12 months, do you remember receiving any information about avoiding unnecessary prescribing OR administering OR dispensing of antibiotics?
- If yes, how did you first get this information about avoiding unnecessary prescribing OR administering OR dispensing of antibiotics?
- Did the information contribute to changing your views about avoiding unnecessary prescribing OR administering OR dispensing of antibiotics?
- Which source(s) of information has had the most influence on changing your views?
- On the basis of the information you received, have you changed your practice on prescribing OR administering OR dispensing of antibiotics?
- If yes, please list what has had the most influence on changing your practice?
- If no, why not?

Section 7 - Campaign and training

- At what level do you think it is most effective to tackle resistance to antibiotics?
- What initiatives are you aware of in your country which focus on antibiotic awareness and resistance?
- To what extent do you agree or disagree with the following statements regarding the national initiatives about prudent use of antibiotics in your country?
    - There has been good promotion of prudent use of antibiotics and antibiotic resistance in my country
    - I believe the national campaign has been effective in reducing unnecessary antibiotic use and controlling antibiotic resistance
- Does your country have a national action plan on antimicrobial resistance?
- Have you heard of European Antibiotic Awareness Day (EAAD) or World Antibiotic Awareness Week (WAAW)?
- How effective do you believe EAAD and WAAW have been in raising awareness about prudent use of antibiotics and antibiotic resistance in your country?
- On which topics would you like to receive more information?

Section 8 - Future Contact

- How did you find out about the survey?
- May we contact you in the future about:
    - Your survey responses
    - Other relevant AMR activities
- Please provide your name
- Please provide your email address

The PDF of the online questionnaire is available as supplement 3

Section 9 - Question for prescribers

- **Do you currently prescribe antibiotics or are you currently an undergraduate health student?**
- **How often do you prescribe antibiotics?**
- **To what extent do you agree or disagree with the following statements?**
    - I am confident making antibiotic prescribing decisions
    - I have confidence in the antibiotic guidelines available to me
    - I have a key role in helping control antibiotic resistance
    - I consider antibiotic resistance when treating a patient
    - I have easy access to antibiotic guidelines I need to treat infections
    - I feel supported to not prescribe antibiotics when they are not necessary
- **Considering the last one week only:**
    - How often would you have preferred not to prescribe an antibiotic but were not able during the last one week?
    - How often did the fear of patient deterioration or fear of complications lead you to prescribe antibiotics during the last one week?
    - How often did you prescribe antibiotics because it took less time than to explain the reason why they are not indicated during the last one week?
    - How often did you stop an antibiotic prescription earlier than the prescribed course length during the last one week?
    - How often did you prescribe antibiotics in situations in which it is impossible for you to conduct a follow-up of the patient during the last one week?
    - How often did you prescribe an antibiotic to maintain the relationship with the patient during the last one week?
    - How often did you prescribe an antibiotic because you were uncertain about the diagnosis of infection during the last one week?
    - How often did you prescribe a shorter course of treatment as compared to available guidelines during the last one week?
    - How often did you discontinue early (within three days after initiation) a treatment because bacterial infection was not likely after all during the last one week?
- **What strategies do you employ to prescribe antibiotics prudently?**

Section 10 – Demographic questions asked to each respondent at the beginning of the survey

- Are you involved in diagnosis, prescribing, clinical checking prescriptions, dispensing, administration, or provision of advice of antibiotics to patients or members of the public?
- In what country do you currently practice?
- Please specify which continent.
- Please specify in which country you practice.
- What is your core profession?
- What is your predominant role? (i.e.>50% of your time)
- Where do you predominantly practice? (i.e. >50% of your time)
- How many years have you been practicing in your current profession?
- What is your age?
- What gender do you most identify with?
- Which of the following social media networks do you mainly use for professional activities?
- In your current role are you contributing to/leading antimicrobial stewardship programmes or tackling AMR?
