# Supplementary material for: Healthcare workers’ knowledge, attitudes and behaviours with respect to antibiotics, antibiotic use and antibiotic resistance across 30 EU/EEA countries in 2019
Source: Euro Surveill. 2021 Mar 25;26(12):1900633. doi: 10.2807/1560-7917.ES.2021.26.12.1900633 (PMC7995558; doi:10.2807/1560-7917.ES.2021.26.12.1900633)
Supplement: Supplementary Material 3 [file 1900633_Supplementary_material_3.pdf]

This supplementary material is hosted by Eurosurveillance as supporting information alongside the article 'Healthcare workers' knowledge, attitudes and behaviours with respect to antibiotics, antibiotic use and antibiotic resistance across 30 EU/EEA countries in 2019', on behalf of the authors, who remain responsible for the accuracy and appropriateness of the content. The same standards for ethics, copyright, attributions and permissions as for the article apply. Supplements are not edited by Eurosurveillance and the journal is not responsible for the maintenance of any links or email addresses provided therein.

Supplement 3: CHERRIES checklist for web-based studies[1]

Item category	Checklist item	Description
Design		The target population was a convenience sample of healthcare workers across 30 EU/EEA member states. A quota sampling approach was used to determine the minimum survey sample size required in each of the participating countries.
Ethics	Ethics approval	Ethical requirements for ECDC are determined at the level of the EU Treaties, the Charter of Fundamental Rights, and international conventions such as the Declaration of Helsinki. These were duly followed throughout the study.
	Informed consent	All respondents participated strictly in their professional capacity, and their participation in the survey was in all cases based on informed consent. They were informed of the purpose of the study and that participation was voluntary and that their responses were anonymous. On the front page which included the introduction they were also asked to confirm if they were involved in diagnosis, prescribing, clinical checking prescriptions, dispensing, administration, or provision of advice of antibiotics to patients or members of the public. Consent was indicated when respondents clicked next at the bottom of the introductory page and moved to the next page.<br><br>An estimate time (5 – 10 minutes) to complete the survey (based on the pilot phase) was also provided on the survey introduction. Responses to questions were stored as well as completion data, start and end time.<br><br>Also, on the introductory page participants were provided with the study lead's name and email address
	Data protection	Proprietary survey software and local servers were used to ensure data protection.<br>Some participants provided their email address if they wished to be contacted in the future in relation to the survey. This was not linked to survey results. The data collected through the survey tool is not accessible without account permissions to view the responses. The fully de-identified dataset was used for analysis and is kept on password protected computers.
Development and pre-testing		In October 2018, the ECDC National Focal Points for Antimicrobial Resistance and the ECDC National Focal Points for Communication of European Union (EU) Member States and two European Economic Area (EEA) countries (Iceland and Norway), as well as selected European health professional organisations or groups, were invited to designate country representatives to participate in the study as members of a Project Advisory Group (PAG). The PAG comprised 87 individuals representing EU/EEA countries and European professional organisations that are listed at the end of the article.

		The initial phase of developing the survey tool involved reviewing the literature using specified search terms. The draft survey tool then went through an iterative process of two rounds of Delphi consensus requiring members of the PAG to assess the relevance and comment on the proposed questions. Following the two rounds, the online survey tool was piloted by 224 healthcare professionals and workers across 27 countries for additional comments on the content, interpretation of questions and time-scale for completion. For the next stage, feedback from the pilot was addressed, and explanations for updates to the survey were shared with the PAG to develop a consensus that the survey was appropriate for assessing healthcare professionals and workers' knowledge and attitudes on antibiotics and antibiotics resistance across Europe. Following the consensus process and pilot, the final survey including 43 questions was finalised. The survey was translated into 25 languages, which was then shared with members of the PAG to review and assess any divergences from the validated questions (appendix 1).
Recruitment process	Open vs closed survey	This was an open survey.
	Contact mode	When the survey tool was finalised, an email was sent to PAG members for cascade through their relevant channels to healthcare workers. PAG members are members of national/international organisations and institutions. The survey link was also added to the ECDC website and promoted via social media. Twitter was the primary social media tool used by the project team adding hashtag #ECDCAntibioticSurvey. It is however possible that PAG members and member countries used other relevant social media outlets or communication methods e.g. newsletter to members. (Appendix 2 and 3)
	Advertising the survey	The project team at PHE developed relevant communications including template emails and webpage advertorials for promoting the survey and to ensure consistency of messaging (Appendix 1). The link to the survey was advertised through the ECDC webpage, communications cascaded via the PAG group, and Twitter was used as the primary social media tool by various organisations to promote the survey. Individual country focal points were able to use their relevant online mailing lists which consisted of any healthcare workers.

Item category	Checklist item	Description
Survey administration	Web/email	This was a web-based survey. Responses were collected through the online survey platform and stored on secure local servers. Responses were multiple choice, numeric, and open text.
	Context	Signpost to the survey URL was available on ECDC website as well as organisation and institutions/countries represented on the project advisory group. The main audience for these webpages will be health or public health workers, in particular country representatives or national leads.<br><br>https://antibiotic.ecdc.europa.eu/en/healthcare-workers/survey-healthcare-workers-knowledge-and-attitudes-about-antibiotics-and
	Mandatory/voluntary	Voluntary.
	Incentives	There were no incentives offered to individuals.
	Time/date	The data was collected from *31st January to 4th March 2019 (following a two extension from initial deadline advertised).*
	Item randomisation	The items were not randomised or alternated – the same order of questions was received by all participants.
	Adaptive questioning	Some questions and pages were conditionally displayed e.g. specific questions for those who stated that they prescribed antibiotics as part of their role.
	Number of items	There were 43 items displayed across 9 pages. The number of items per page varied from 1 question to 12. Due to the adaptive nature of the questionnaire, not all respondents were presented with all items e.g. the questions/page specific to prescribers.
	Number of screens	The full survey was distributed over 9 pages
	Completeness check	Several questions were set as requiring a response especially the adaptive questions, however these also included a Don’t know/unsure option Non-response options were provided.

Item category	Checklist item	Description
	Review step	Respondents were able to review and change their answers using a ‘back’ button before submitting their form
Response rates	Unique site visitor	IP addresses and cookies were not recorded to ensure confidentiality. Also it was expected that users were likely to respond from their workplace, where there were likely to be using shared devices.
	View rate	The survey URLs (across all languages) were clicked on 70, 747 times. The English survey had the most clicks 21, 506, followed by Italian (8154) and Spanish (8307).
	Participation rate	In total, 18 365 healthcare workers from the 30 EU/EEA countries responded to the survey; thus exceeding the estimated required overall quota size of 11 931 respondents. The number of responses per country and profession and the target minimum quota sample size is presented in Supplementary Table 1. Twenty-four (80%) countries achieved or exceeded the quota sample size of respondents, while four countries received less than 60% of the quota sample size. The number of responses from doctors, dentists and pharmacists significantly exceeded the quota sample size for these professions (Supplementary 2_ Table 1). The number of responses from the nursing profession was only slightly higher than the quota sample size, and only 55% of the quota sample size for other healthcare workers was achieved.
	Completion rate	Of the 25, 572 provided a response to the question on the introductory page and accessed the main part of section of the survey, 18 365 healthcare workers from the 30 EU/EEA countries responded to the first non-demographic question and subsequently included for analysis (72%);
Preventing multiple entries from same individual	Cookies used	No
	IP check	No
	Log file analysis	Not used
	Registration	Entry to the survey was via a unique login provided to each invitee to the survey.
Analysis	Handling of incomplete questionnaires	Only responses from respondents that completed the first question on page 3 (testing knowledge) were included for analysis, irrespective of completeness of full survey. Page 1 was the introductory page and included on qualifying question and page 2 questions were demographic questions only and did not assess any of the survey objectives.
	Questionnaires with atypical timestamp	There was no timeframe set or used as a cut-off point
	Statistical correction	No statistical correction was made during analysis

Eysenbach G. Improving the quality of web surveys: The Checklist for Reporting Results of Internet E-Surveys (CHERRIES). J Med Internet Res. 2004;6(3):1–6.

COMMUNICATIONS

Launch of Europe wide survey of healthcare workers knowledge and attitudes about antibiotic use and resistance

On 28 January 2019, an ECDC-funded* survey to assess healthcare workers' knowledge and perceptions about antibiotic use and resistance launched across Europe. Previous studies have mostly focused on the general public and medical students, highlighting a gap in the understanding of these topics by healthcare workers and by other health students.

Following a process of validation and piloting across Europe, the survey is now available for completion. **The study closes on 14 February 2019.**

Click below to access the English version (EN):
https://surveys.phe.org.uk/TakeSurvey.aspx?SurveyID=9IKJ5585H

The survey is also available in 25 additional languages (Table 1).

The aim is to have a return of 10,000+ responses with representation from healthcare workers including doctors, nurses, midwives, dentists, pharmacists, clinical scientists, hospital managers, allied health professionals, nursing associates, technicians and healthcare students. Table 2 highlights the calculated sample size per professional group by country however we would welcome significantly more responses.

The objectives of the study for ECDC are:

- to gain a better understanding of their knowledge and perceptions to provide a base to support future needs in terms of policy and education changes, and
- to fill in gaps in terms of evaluation of communication campaigns targeting healthcare workers

Please cascade the link of the survey actively to relevant organisations and colleagues as well as healthcare students. If you are using social media please use **#ECDCAntibioticSurvey.** For questions about this survey do not hesitate to contact Dr Diane Ashiru-Oredope.

Dr Diane Ashiru-Oredope
Project Lead; Europe wide Survey of healthcare workers knowledge and attitudes about antibiotics and antibiotic resistance
espaur@phe.gov.uk

* European Centre for Disease Prevention and Control

#ECDCAntibioticSurvey

ECDC Europe wide survey of healthcare workers knowledge and attitudes about antibiotic use and resistance

Table 1. ECDC Antibiotic Survey - Language Links

Language Codes	Language	Survey Link:
BG	Bulgarian	https://surveys.phe.org.uk/TakeSurvey.aspx?SurveyID=92KJ497KH
CS	Czech	https://surveys.phe.org.uk/TakeSurvey.aspx?SurveyID=94KJ4m3MH
DA	Danish	https://surveys.phe.org.uk/TakeSurvey.aspx?SurveyID=94KJ4m8MH
DE	German	https://surveys.phe.org.uk/TakeSurvey.aspx?SurveyID=96KJ4n31H
EL	Greek	https://surveys.phe.org.uk/TakeSurvey.aspx?SurveyID=96KJ4n41H
ES	Spanish	https://surveys.phe.org.uk/TakeSurvey.aspx?SurveyID=98KJ4nl3H
ET	Estonian	https://surveys.phe.org.uk/Deploy.aspx?SurveyID=m2KJ4nmKH
FI	Finnish	https://surveys.phe.org.uk/TakeSurvey.aspx?SurveyID=98KJ4o23H
FR	French	https://surveys.phe.org.uk/TakeSurvey.aspx?SurveyID=92KJ496KH
GA	Gallic	https://surveys.phe.org.uk/TakeSurvey.aspx?SurveyID=92KJ49lKH
HR	Croatian	https://surveys.phe.org.uk/TakeSurvey.aspx?SurveyID=92KJ4l4KH
HU	Hungarian	https://surveys.phe.org.uk/TakeSurvey.aspx?SurveyID=94KJ4lmMH
IS	Icelandic	https://surveys.phe.org.uk/TakeSurvey.aspx?SurveyID=94KJ4m4MH
IT	Italian	https://surveys.phe.org.uk/TakeSurvey.aspx?SurveyID=94KJ4n2MH
LT	Lithuanian	https://surveys.phe.org.uk/TakeSurvey.aspx?SurveyID=98KJ4o43H
LV	Latvian	https://surveys.phe.org.uk/TakeSurvey.aspx?SurveyID=98KJ4o53H
MT	Maltese	https://surveys.phe.org.uk/TakeSurvey.aspx?SurveyID=98KJ4o73H
NL	Dutch	https://surveys.phe.org.uk/TakeSurvey.aspx?SurveyID=94KJ4m2MH
NO	Norwegian	https://surveys.phe.org.uk/TakeSurvey.aspx?SurveyID=98KJ4om3H
PL	Polish	https://surveys.phe.org.uk/TakeSurvey.aspx?SurveyID=9IKJ4pl5H
PT	Portuguese	https://surveys.phe.org.uk/TakeSurvey.aspx?SurveyID=98KJ4p23H
RO	Romanian	https://surveys.phe.org.uk/TakeSurvey.aspx?SurveyID=9IKJ4p85H
SK	Slovak	https://surveys.phe.org.uk/TakeSurvey.aspx?SurveyID=9IKJ4pm5H
SL	Slovenian	https://surveys.phe.org.uk/TakeSurvey.aspx?SurveyID=9IKJ5565H
SV	Swedish	https://surveys.phe.org.uk/TakeSurvey.aspx?SurveyID=9IKJ5595H
EN	English	https://surveys.phe.org.uk/TakeSurvey.aspx?SurveyID=9IKJ5585H

#ECDCAntibioticSurvey

Appendix 2: Sample advert on a professional body’s webpage and newsletter to members

Members area

Joint Action Antimicrobial Resistance and Healthcare-Associated Infections

About us | Our work | Get Involved | News | Results | Contact us

ECDC Europe wide survey of healthcare workers knowledge and attitudes about antibiotic use and resistance

The European Centre for Disease Prevention and Control (ECDC) has launched a **survey to assess healthcare workers’ knowledge and perceptions about antibiotic use and resistance**. Previous studies have mostly focused on the general public and medical students, highlighting a gap in the understanding of these topics by healthcare workers and by other health students.

Following a process of validation and piloting across Europe, the survey is now available for completion. The aim is to have a return of 10,000+ responses with representation from healthcare workers including doctors, nurses, midwives, dentists, pharmacists, clinical scientists, hospital managers, allied health professionals, nursing associates, technicians and healthcare students.

The objectives of the study for ECDC are:

- to gain a better understanding of their knowledge and perceptions to provide a base to support future needs in terms of policy and education changes, and
- to fill in gaps in terms of evaluation of communication campaigns targeting healthcare workers

To complete the survey in English click here

The survey is also available in 25 additional languages (to access them click here)

Deadline: 14 February 2019

If you share this survey via social media please use #ECDCAntibioticSurvey

For more info please contact:
Dr Diane Ashiru-Oredope
Project Lead; Europe wide Survey of healthcare workers knowledge and attitudes about antibiotics and antibiotic resistance
espaur@phe.gov.uk

Share

Appendix 3: Sample social media messages promoting the survey URL

<table>
<tr>
<td>

Robert Koch-Institut
@rki_de

Gesucht: Ärzte, Krankenpfleger, Apotheker, Zahnärzte, Studenten und alle anderen, die im Gesundheitswesen tätig sind. Jetzt teilnehmen am #ECDCAntibioticSurvey

Testen Sie Ihr Wissen zu #Antibiotikaresistenzen: surveys.phe.org.uk/TakeSurvey.asp…

@ECDC_EU #AMR #AntibioticResistance

Translate Tweet

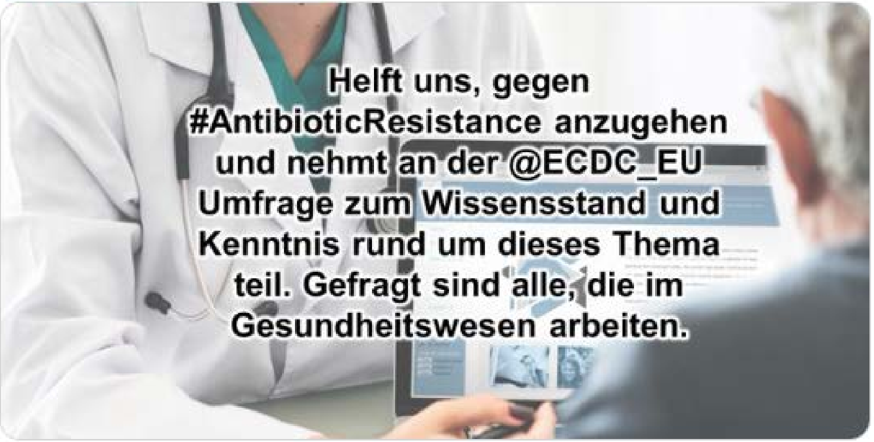

7:57 am · 22 Feb 2019 · Twitter Web Client

</td>
<td>

NICE
@NICEComms

The ECDC survey of healthcare workers knowledge and attitudes about antibiotic use and resistance is now live #ECDCAntibioticSurvey. Take 10 minutes and help to achieve the target of 10,000+ responses: bit.ly/2G5cJhJ

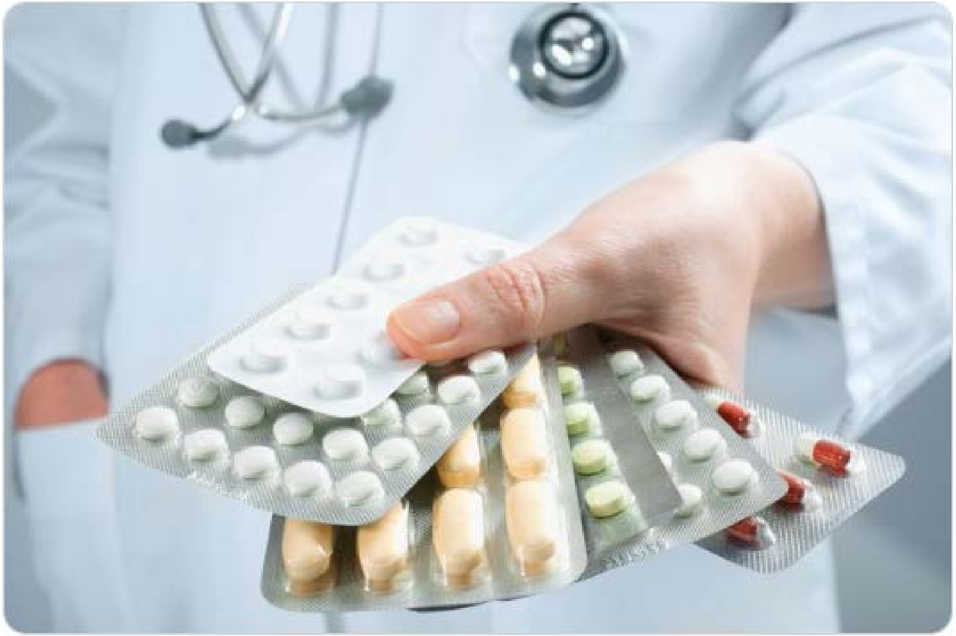

2:00 pm · 4 Feb 2019 · TweetDeck

</td>
</tr>
<tr>
<td></td>
<td></td>
</tr>
</table>

Newcastle Hospitals @NewcastleHosps

Have you completed the #ECDCAntibioticSurvey by @PHE_uk and @ECDC_EU? They looking to understand healthcare workers knowledge and attitudes about antibiotic use and resistance. Survey closes Thursday 14 Feb: surveys.phe.org.uk/TakeSurvey.asp...

2:47 pm · 8 Feb 2019 · Twitter Web Client

Healthcare Infection Society @HIS_infection

The ECDC and PHE are seeking responses from healthcare workers
about their knowledge of and attitudes towards antibiotic use and resistance. Take the survey here: surveys.phe.org.uk/TakeSurvey.asp... The survey closes 14 February 2019. #ECDCAntibioticSurvey

8:50 am · 12 Feb 2019 · TweetDeck

ECDC @ECDC_EU

Extended deadline: Take part in the #ECDCAntibioticSurvey by 1 March 2019 to help us gain an understanding of European #healthcare worker's knowledge & perceptions about #antibiotics & #AntibioticResistance.

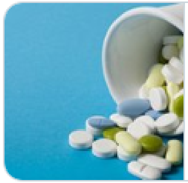

Survey of healthcare workers' knowledge and attitudes about ...
On 28 January, Public Health England (PHE) launched a new multilingual survey, funded by the European Centre for Diseas...
ecdc.europa.eu

10:31 am · 25 Feb 2019 · Twitter Web Client

41 Retweets **25** Likes

European Health Management Association @EHMAinfo

Do not miss the #ECDCAntibioticSurvey ! @ECDC_EU

Diane Ashiru, PhD @DrDianeAshiru · 30 Jan 2019
>2,700 responses already to the #ECDCAntibioticSurvey . Huge thanks to everyone who has shared. Please continue to encourage ALL healthcare colleagues and health students to complete. The more responses there are, the more useful the data will be. surveys.phe.org.uk/TakeSurvey.asp...

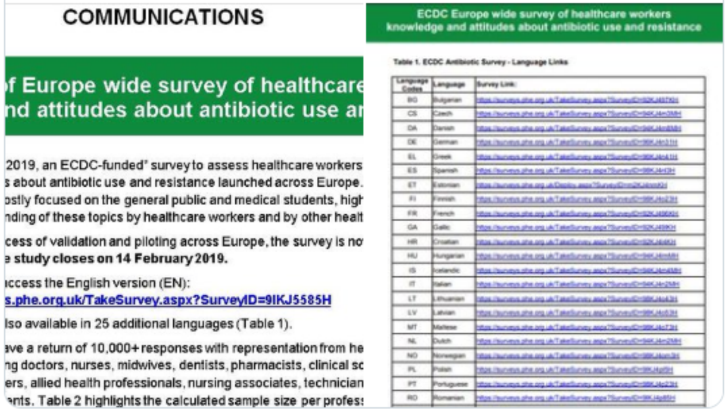

COMMUNICATIONS

ECDC Europe wide survey of healthcare workers knowledge and attitudes about antibiotic use and resistance

f Europe wide survey of healthcare
nd attitudes about antibiotic use ar

2019, an ECDC-funded' survey to assess healthcare workers
s about antibiotic use and resistance launched across Europe.
ostly focused on the general public and medical students, high
nding of these topics by healthcare workers and by other healt
cess of validation and piloting across Europe, the survey is no
e study closes on 14 February 2019.

ccess the English version (EN):
s.phe.org.uk/TakeSurvey.aspx?SurveyID=9IKJ5585H

lso available in 25 additional languages (Table 1).

ave a return of 10,000+ responses with representation from he
ng doctors, nurses, midwives, dentists, pharmacists, clinical sc
ers, allied health professionals, nursing associates, technician
ents. Table 2 highlights the calculated sample size per profes

Table 1. ECDC Antibiotic Survey - Language Links

4:14 pm · 30 Jan 2019 · TweetDeck
