# Supplementary material for: Healthcare workers’ knowledge, attitudes and behaviours with respect to antibiotics, antibiotic use and antibiotic resistance across 30 EU/EEA countries in 2019
Source: Euro Surveill. 2021 Mar 25;26(12):1900633. doi: 10.2807/1560-7917.ES.2021.26.12.1900633 (PMC7995558; doi:10.2807/1560-7917.ES.2021.26.12.1900633)
Supplement: Supplementary Material 4 [file 1900633_Supplementary_material_4.pdf]

This supplementary material is hosted by Eurosurveillance *as supporting information alongside the article 'Healthcare workers' knowledge, attitudes and behaviours with respect to antibiotics, antibiotic use and antibiotic resistance across 30 EU/EEA countries in 2019', on behalf of the authors, who remain responsible for the accuracy and appropriateness of the content. The same standards for ethics, copyright, attributions and permissions as for the article apply. Supplements are not edited by* Eurosurveillance *and the journal is not responsible for the maintenance of any links or email addresses provided therein.*

Supplement 4:

Healthcare workers' knowledge, attitudes and behaviours with respect to antibiotics, antibiotic use and resistance in 30 EU/EEA countries: supplementary tables and figures

Supplementary Table 1: Number of responses and quota sample size by country and profession, by country, EU/EEA

Country	Physicians*		Nurses, nursing professionals & midwives		Dentists		Pharmacists		Other healthcare workers		Responses from healthcare workers without quota sample size	Total	
	Quota sample size	Total responses	Quota sample size	Total responses	Quota sample size	Total responses	Quota sample size	Total responses	Quota sample size	Total responses		Number required based on quota sample size	Total responses
Austria	90	366	71	274	10	87	12	200	37	95	103	220	1 125
Belgium	70	177	126	69	17	14	28	86	48	33	20	288	399
Bulgaria	59	7	34	1	16		13	22	24	1	0	146	31
Croatia	27	40	28	2	7	9	6	18	14	0	5	81	74
Cyprus	6	65	5	32	2		2	6	3	5	0	17	108
Czech Republic	78	936	89	4	16	1	14	5	39	6	12	236	964
Denmark	42	251	98	253	8	119	6	55	31	45	50	185	773
Estonia	9	35	8	37	3		2	48	4	5	3	26	128
Finland	35	81	80	457	8	39	12	120	27	51	22	162	770
France	419	376	704	203	86	11	140	224	270	36	20	1 619	870
Germany	690	151	1 081	28	141	7	105	200	403	3	17	2 419	406
Greece	142	38	38	20	26	15	23	136	46	3	9	274	221
Hungary	63	105	65	187	12		15	11	31	46	29	186	378
Iceland	3	3	5	7	1	31	0	2	2	1	0	10	44
Ireland	28	63	61	14	6	24	11	25	21	6	5	127	137
Italy	479	890	354	276	97	131	140	700	214	82	88	1 285	2 167
Latvia	13	138	9	5	3	89	3	3	6	4	3	34	242
Lithuania	26	80	23	34	6	1	7	2	12	11	9	73	137
Luxembourg	3	7	7	16	1		1	5	2	6	2	15	36
Malta	3	6	4	10	0	10	1	9	2	21	1	11	57
Netherlands	119	119	184	7	19	48	7	7	66	0	9	395	190
Norway	47	616	94	630	9	38	8	35	32	88	59	191	1 466

Poland	177	363	220	193	25	92	56	259	96	81	134	574	1 122
Portugal	99	74	69	87	20	19	18	184	41	11	11	247	386
Romania	112	314	135	158	33	18	34	68	63	15	14	376	587
Slovakia	38	221	20	179	5	1	8	27	14	8	0	86	436
Slovenia	12	60	20	10	3	9	3	8	8	3	5	46	95
Spain	355	1 080	301	310	71	247	112	210	168	18	27	1 008	1 892
Sweden	84	262	116	307	16	3	15	60	46	48	40	276	720
United Kingdom	365	427	548	962	70	22	113	523	219	360	110	1 315	2 404
**EU/EEA**	**3 692**	**7 351**	**4 599**	**4 772**	**735**	**1 085**	**915**	**3 258**	**1 988**	**1 092**	**807**	**11 929**	**18 65**

* The term physician is defined by the European statistical office (Eurostat) [18] as including generalist medical practitioners (general practitioners (GPs) and other generalist medical practitioners) and specialist medical practitioners (medical specialists and surgical specialists). For the purposes of this survey we chose to use the term medical doctor, and defined it in the questionnaire list as including: general practice, surgeon, specialists - public health, microbiologist and infectious disease physician.

Supplementary Table 2: Percentage of respondents who agreed with the statements: "I know what antibiotic resistance is" and "I have sufficient knowledge about how to use antibiotics appropriately for my current practice", by country, EU/EEA

	**I know what antibiotic resistance is**		**I have sufficient knowledge about how to use antibiotics appropriately for my current practice**	
**Country**	Number answering question	Agree or strongly agree (%)	Number answering question	Agree or strongly agree (%)
Austria	1 125	92	1 124	75
Belgium	399	97	399	78
Bulgaria	31	100	30	90
Croatia	74	96	74	85
Cyprus	108	100	108	93
Czech Republic	964	95	964	84
Denmark	773	97	773	79
Estonia	128	98	128	81
Finland	770	98	770	78
France	870	96	870	71
Germany	406	96	406	74
Greece	221	97	221	86
Hungary	378	96	378	80
Iceland	44	93	44	91
Ireland	137	95	137	85
Italy	2 167	97	2 167	86
Latvia	242	98	242	84
Lithuania	137	99	137	81
Luxembourg	36	97	36	61
Malta	57	91	57	63
Netherlands	190	98	190	80
Norway	1 466	94	1 465	74
Poland	1 122	95	1 121	76
Portugal	386	98	386	90
Romania	587	91	586	86
Slovakia	436	95	436	86
Slovenia	95	97	95	74
Spain	1 892	96	1 892	83
Sweden	720	93	719	80
United Kingdom	2 404	96	2 404	78
**EU/EEA**	**18 365**	**96**	**18 359**	**80**

Supplementary Table 3: Average score of those who answered all 7 knowledge questions correctly and percentage of respondents answering all questions correctly, by country, EU/EEA (n=18 348)

Country	Number of respondents who provided and completed all seven key knowledge questions (% total number of survey participants)	Average score (out of 7)	% of respondents answering all questions correctly (7/7)
Austria	1 124 (99.9)	6.17	53
Belgium	399 (100.0)	6.38	59
Bulgaria	31 (100.0)	6.16	48
Croatia	74 (100.0)	6.58	73
Cyprus	108 (100.0)	6.34	61
Czech Republic	963 (99.9)	6.48	62
Denmark	773 (100.0)	6.24	49
Estonia	127 (99.2)	5.87	40
Finland	769 (99.9)	6.48	64
France	870 (100.0)	6.58	69
Germany	406 (100.0)	6.55	68
Greece	221 (100.0)	6.17	48
Hungary	378 (100.0)	6.02	46
Iceland	44 (100.0)	6.43	61
Ireland	137 (100.0)	6.61	71
Italy	2 167 (100.0)	6.19	50
Latvia	239 (98.8)	5.82	41
Lithuania	137 (100.0)	6.56	66
Luxembourg	36 (100.0)	6.06	53
Malta	57 (100.0)	6.04	47
Netherlands	190 (100.0)	6.42	57
Norway	1 465 (99.9)	6.40	59
Poland	1 118 (99.6)	6.48	68

Portugal	385 (99.7)	6.33	53
Romania	586 (99.8)	6.24	56
Slovakia	436 (100.0)	6.15	48
Slovenia	95 (100.0)	6.39	51
Spain	1 892 (100.0)	6.49	65
Sweden	718 (99.7)	6.38	55
United Kingdom	2 403 (100.0)	6.36	59
**EU/EEA**	**18 348 (99.9)**	**6.35**	**58**

Supplementary Table 4: Average score on the seven key knowledge questions per professional group and percentage of respondents of each profession who achieved all correct answers (7/7), EU/EEA (n=18 365)

Profession	Number of participants in the survey	Number of respondents answering all seven key knowledge questions (% of total number of participants)	Average score	% of respondents answering all questions correctly
Medical doctor	7 351	7 350 (100.0)	6.56	68
Scientist	461	461 (100.0)	6.47	64
Pharmacist	3 258	3 256 (99.9)	6.41	59
Nurse	4 312	4 307 (99.9)	6.22	51
Dentist	1 085	1 082 (99.7)	6.18	50
Midwife	210	209 (99.5)	6.24	49
Other healthcare worker	176	175 (99.4)	5.85	41
Unknown	146	143 (97.9)	5.54	40
Pharmacy Technician	250	250 (100.0)	6.03	40
Allied Health Professional	633	632 (99.8)	5.88	38
Dental care professional	33	33 (100.0)	5.61	33
Nursing associate/assistant	250	250 (100.0)	5.58	30
Other	200	200 (100.0)	5.43	29
**All healthcare workers**	**18 365**	**18 348 (99.9)**	**6.35**	**58**

Capability – knowledge test (One health)

When asked to what extent a number of environmental and animal health factors are important to contributing to antibiotic resistance in bacteria for humans, the majority (89%) of respondents agreed or strongly agreed that excessive use of antibiotics in livestock and food production contributes to antibiotic resistance in humans; however, only about two-thirds (63%) agreed or strongly agreed that environmental factors such environmental waste water was a contributing factor to antimicrobial resistance (AMR) in humans. Scientists had the highest number of respondents strongly agree or agree that environmental factors such as waste water in the environment can contribute to antibiotic resistance in bacteria in humans.

Supplementary Figure 1: Proportion of respondents who agreed or strongly agreed that animal health and environmental factors are important in contributing to antibiotic resistance in bacteria from humans, EU/EEA

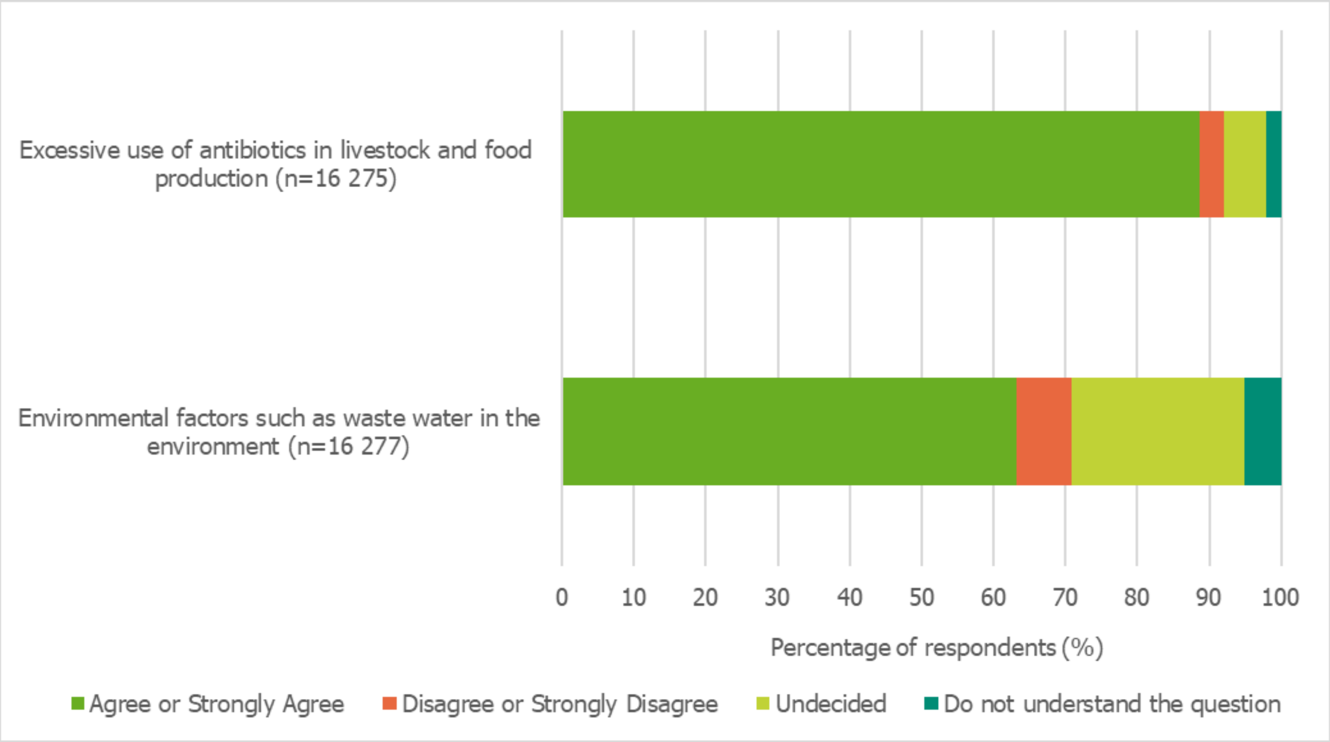

Only 27% (n=4998) of respondents knew that it is illegal to use antibiotics to stimulate growth in farm animals in the EU, most were either unsure (44%, n=8054) or believed this to be legal practice (29%, n=5291)).

Capability – Knowledge test (Hand hygiene)

Across the EU/EEA countries there was variation in the percentage of respondents who reported that they could list the World Health Organization (WHO) five moments of hand hygiene (56%; range: 29% to 78%). However, more respondents across the 30 countries (EU average 87%) agreed they need to perform hand hygiene as often as recommended (i.e. ‘I perform hand hygiene (as often as recommended) if I had gloves on when in contact with patients or biological material’. Nurses and nursing associates/technicians were the professions most aware of the WHO’s five moments of hand hygiene (73%) and most likely to perform hand hygiene if gloves had contacted patients or biological material (96% and 92%, respectively).

Supplementary Figure 2: Percentage of respondents who stated they could list the WHO’s five moments for hand hygiene and thought they needed to perform hand hygiene even if gloves used as recommended by WHO, EU/EEA

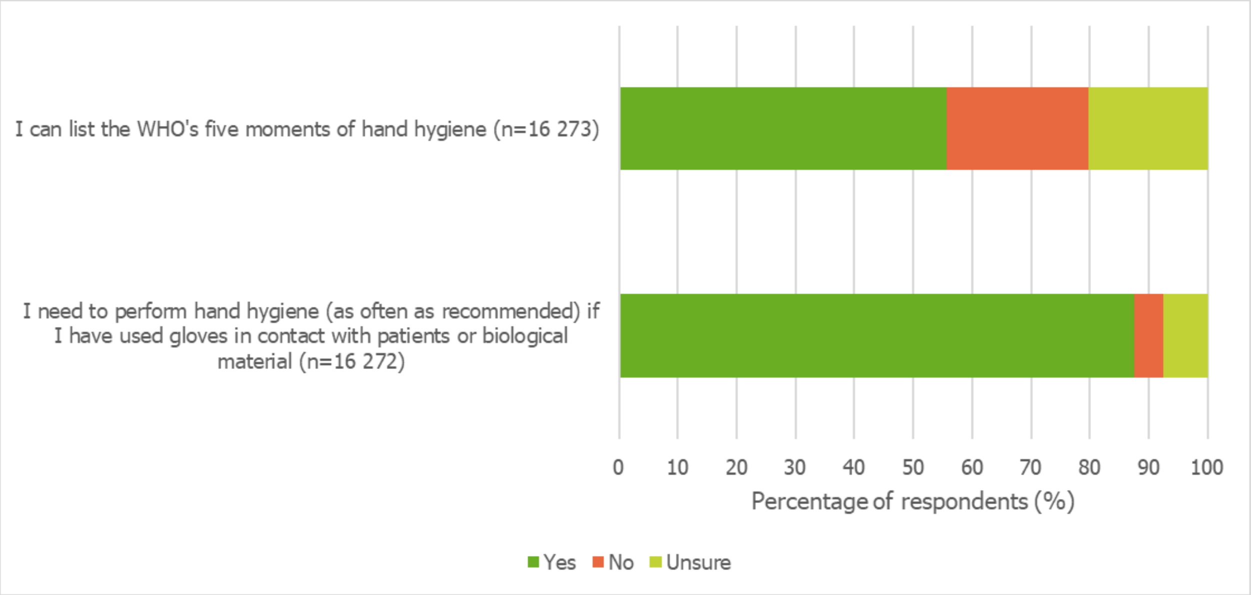

Supplementary Table 5: Percentage of respondents with direct patient/public involvement who agreed or disagreed that they have easy access to guidelines they need on managing infections, by country, EU/EEA (n=14 301)

Country	Agree or Strongly Agree (%)	Disagree or Strongly Disagree (%)	Undecided (%)	Do not understand the question (%)	N/A (%)
Austria (n=769)	71	11	14	1	3
Belgium (n=312)	72	7	13	4	4
Bulgaria (n=23)	70	4	26	0	0
Croatia (n=57)	63	21	12	0	4
Cyprus (n=91)	78	10	12	0	0
Czech Republic (n=828)	85	8	5	1	1
Denmark (n=613)	82	5	9	0	4
Estonia (n=98)	79	7	12	0	2
Finland (n=632)	64	15	17	0	4
France (n=719)	84	8	6	0	2
Germany (n=365)	84	7	8	0	1
Greece (n=188)	67	5	27	1	1
Hungary (n=218)	79	5	15	1	1
Iceland (n=36)	78	11	11	0	0
Ireland (n=115)	82	8	9	0	2
Italy (n=1721)	61	16	18	0	5
Latvia (n=199)	53	14	23	6	5
Lithuania (n=95)	80	12	8	0	0
Luxembourg (n=23)	65	17	9	0	9
Malta (n=32)	72	13	16	0	0
Netherlands (n=129)	73	5	12	2	8
Norway (n=1040)	83	6	9	0	3
Poland (n=922)	69	12	16	0	3
Portugal (n=333)	66	10	20	1	3
Romania (n=439)	70	11	14	1	6
Slovakia (n=302)	61	21	12	2	5
Slovenia (n=78)	64	10	26	0	0
Spain (n=1606)	77	5	14	1	3
Sweden (n=542)	79	6	13	0	3
United Kingdom (n=1776)	88	5	6	0	2
**EU/EEA (n=14 301)**	**75**	**9**	**12**	**1**	**3**

Supplementary Table 6: Percentage of respondents who agreed or disagreed with the statement "I have easy access to guidelines I need on managing infections", by profession and setting, EU/EEA (n=17 283)

Profession	Setting	Agree or Strongly Agree (%)	Disagree or Strongly Disagree (%)	Undecided (%)	Not applicable (%)	Do not understand (%)
**Allied health professional (n=578)**	Hospital (n=289)	36.3	11.1	14.2	36.7	1.7
	Community (n=171)	32.7	19.9	17.0	30.4	0.0
	Other Settings (n=118)	41.5	17.8	16.1	24.6	0.0
**Dental care professional (n=29)**	Hospital (n=7)	57.1	42.9	0.0	0.0	0.0
	Community (n=6)	50.0	33.3	16.7	0.0	0.0
	Other Settings (n=16)	31.3	18.8	18.8	31.3	0.0
**Dentist (n=1013)**	Hospital (n=92)	59.8	13.0	23.9	3.3	0.0
	Community (n=602)	66.6	11.6	19.4	0.8	1.5
	Other Settings (n=319)	61.8	15.0	18.2	3.1	1.9
**Medical doctor (n=7007)**	Hospital (n=3683)	83.1	6.9	8.5	1.3	0.2
	Community (n=1678)	82.1	7.5	8.9	1.1	0.3
	Other Settings (n=1646)	77.2	7.9	9.8	4.5	0.6
**Midwife (n=196)**	Hospital (n=118)	66.1	12.7	17.8	3.4	0.0
	Community (n=43)	60.5	14.0	14.0	11.6	0.0
	Other Settings (n=35)	71.4	8.6	5.7	11.4	2.9
**Nurse (n=4020)**	Hospital (n=2404)	70.7	9.3	12.9	6.3	0.8
	Community (n=910)	68.6	9.1	13.4	8.2	0.7
	Other Settings (n=706)	65.7	9.5	16.0	8.5	0.3
**Nursing associate/assistant (n=234)**	Hospital (n=136)	54.4	14.0	16.2	14.7	0.7
	Community (n=34)	47.1	23.5	17.6	8.8	2.9
	Other Settings (n=64)	60.9	12.5	12.5	10.9	3.1

**Other (n=181)**	Hospital (n=74)	41.9	4.1	8.1	45.9	0.0
	Community (n=28)	42.9	7.1	14.3	35.7	0.0
	Other Settings (n=79)	45.6	7.6	11.4	35.4	0.0
**Other healthcare worker (n=164)**	Hospital (n=70)	58.6	8.6	11.4	21.4	0.0
	Community (n=29)	31.0	13.8	13.8	41.4	0.0
	Other Settings (n=65)	55.4	7.7	10.8	26.2	0.0
**Pharmacist (n=3078)**	Hospital (n=1152)	84.5	5.2	8.4	1.6	0.3
	Community (n=208)	75.5	10.1	9.1	4.3	1.0
	Other Settings (n=1718)	53.1	16.2	21.0	9.1	0.5
**Pharmacy technician (n=227)**	Hospital (n=94)	67.0	7.4	9.6	16.0	0.0
	Community (n=37)	83.8	5.4	2.7	8.1	0.0
	Other Settings (n=96)	47.9	13.5	22.9	11.5	4.2
**Scientist (n=426)**	Hospital (n=250)	64.4	4.4	8.4	22.8	0.0
	Community (n=7)	28.6	28.6	0.0	42.9	0.0
	Other Settings (n=169)	50.3	9.5	10.7	29.6	0.0
**Unknown (n=130)**	Hospital (n=62)	64.5	4.8	8.1	21.0	1.6
	Community (n=26)	69.2	7.7	7.7	11.5	3.8
	Other Settings (n=42)	42.9	9.5	16.7	31.0	0.0
**All professions (n=17 283)**	Hospital (n=8431)	75.7	7.7	10.4	5.7	0.5
	Community (n=3779)	72.3	9.6	12.2	5.3	0.6
	Other Settings (5073)	62.7	11.9	15.6	9.2	0.7

Supplementary Table 7: Percentage of respondents who agree or disagree with the statement, "I have easy access to the materials I need to give advice on prudent antibiotic use and antibiotic resistance", by profession and setting, EU/EEA (n=17 281)

Profession	Setting	Agree or Strongly Agree (%)	Disagree or Strongly Disagree (%)	Undecided (%)	Not applicable (%)	Do not understand (%)
**Allied health professional (n=578)**	Hospital (n=289)	27.7	13.8	11.8	45.7	1.0
	Community (n=171)	22.8	21.6	19.9	35.7	0.0
	Other Settings (n=118)	32.2	22.0	16.1	29.7	0.0
**Dental care professional (n=29)**	Hospital (n=7)	28.6	28.6	28.6	14.3	0.0
	Community (n=6)	66.7	33.3	0.0	0.0	0.0
	Other Settings (n=16)	25.0	18.8	25.0	31.3	0.0
**Dentist (n=1013)**	Hospital (n=92)	59.8	19.6	19.6	1.1	0.0
	Community (n=602)	54.8	18.3	24.8	1.3	0.8
	Other Settings (n=319)	57.7	16.9	22.3	1.3	1.9
**Medical doctor (n=7007)**	Hospital (n=3683)	69.9	11.6	16.9	1.2	0.3
	Community (n=1678)	69.7	11.5	17.4	0.9	0.5
	Other Settings (n=1646)	70.7	11.7	13.5	3.8	0.2
**Midwife (n=196)**	Hospital (n=118)	45.8	25.4	24.6	3.4	0.8
	Community (n=43)	62.8	11.6	16.3	9.3	0.0
	Other Settings (n=35)	57.1	17.1	14.3	11.4	0.0
**Nurse (n=4019)**	Hospital (n=2403)	58.8	14.5	18.9	7.3	0.5
	Community (n=910)	60.8	13.6	18.2	6.9	0.4
	Other Settings (n=706)	60.5	13.6	18.6	6.7	0.7
**Nursing associate/assistant (n=234)**	Hospital (n=136)	41.9	19.1	16.2	22.1	0.7
	Community (n=34)	41.2	26.5	17.6	14.7	0.0

	Other Settings (n=64)	50.0	15.6	20.3	12.5	1.6
**Other (n=181)**	Hospital (n=74)	32.4	5.4	4.1	58.1	0.0
	Community (n=28)	50.0	14.3	7.1	28.6	0.0
	Other Settings (n=79)	44.3	6.3	15.2	32.9	1.3
**Other healthcare worker (n=164)**	Hospital (n=70)	47.1	10.0	15.7	25.7	1.4
	Community (n=29)	51.7	10.3	3.4	34.5	0.0
	Other Settings (n=65)	46.2	13.8	13.8	24.6	1.5
**Pharmacist (n=3078)**	Hospital (n=1152)	76.6	9.1	12.4	1.6	0.3
	Community (n=208)	75.5	10.6	13.5	0.0	0.5
	Other Settings (n=1718)	66.1	14.6	16.6	2.5	0.3
**Pharmacy technician (n=227)**	Hospital (n=94)	66.0	8.5	12.8	11.7	1.1
	Community (n=37)	75.7	2.7	16.2	5.4	0.0
	Other Settings (n=96)	66.7	9.4	16.7	7.3	0.0
**Scientist (n=425)**	Hospital (n=249)	55.4	4.0	7.6	32.5	0.4
	Community (n=7)	28.6	42.9	0.0	28.6	0.0
	Other Settings (n=169)	53.3	11.8	8.3	26.0	0.6
**Unknown (n=130)**	Hospital (n=62)	62.9	9.7	6.5	21.0	0.0
	Community (n=26)	69.2	7.7	11.5	11.5	0.0
	Other Settings (n=42)	40.5	11.9	19.0	28.6	0.0
**All professions (n=17 281)**	Hospital (n=8429)	64.2	12.3	16.3	6.8	0.4
	Community (n=3779)	62.7	13.6	18.4	4.8	0.5
	Other Settings (n=5073)	63.8	13.5	16.0	6.2	0.5

Supplementary Figure 3: Percentage of respondents who agreed with the following statement, "I have easy access to guidelines I need on managing infections", by country, EU/EEA (n=14 301)

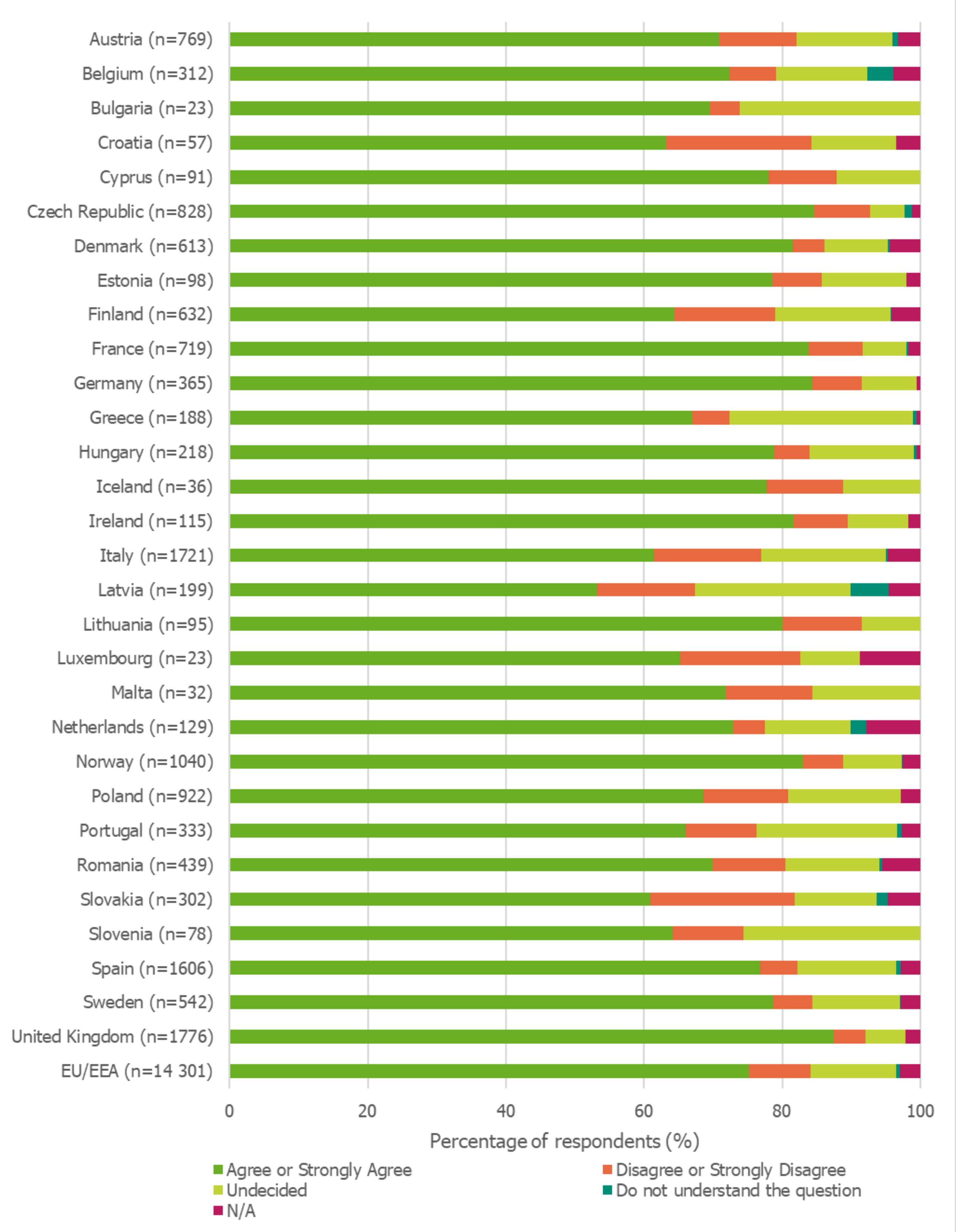

Supplementary Figure 4: Percentage of respondents who agreed with the following statement, "I have good opportunities to provide advice on prudent antibiotic use to individuals", by country, EU/EEA (n=14 296)

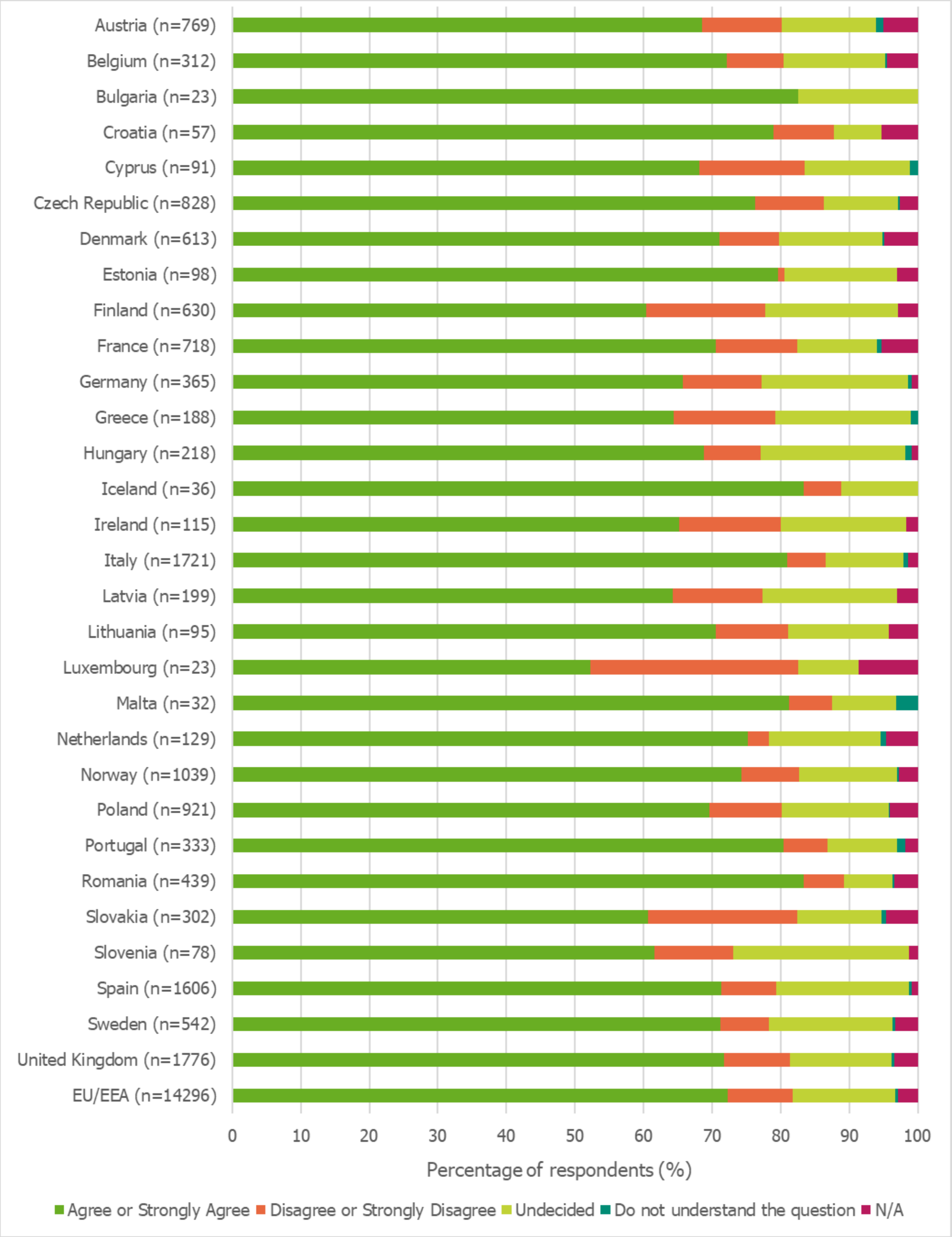

Supplementary Figure 5: Percentage of respondents who agreed with the following statement, "I have easy access to the materials I need to give advice on prudent antibiotic use and antibiotic resistance", by country, EU/EEA (n=14 299)

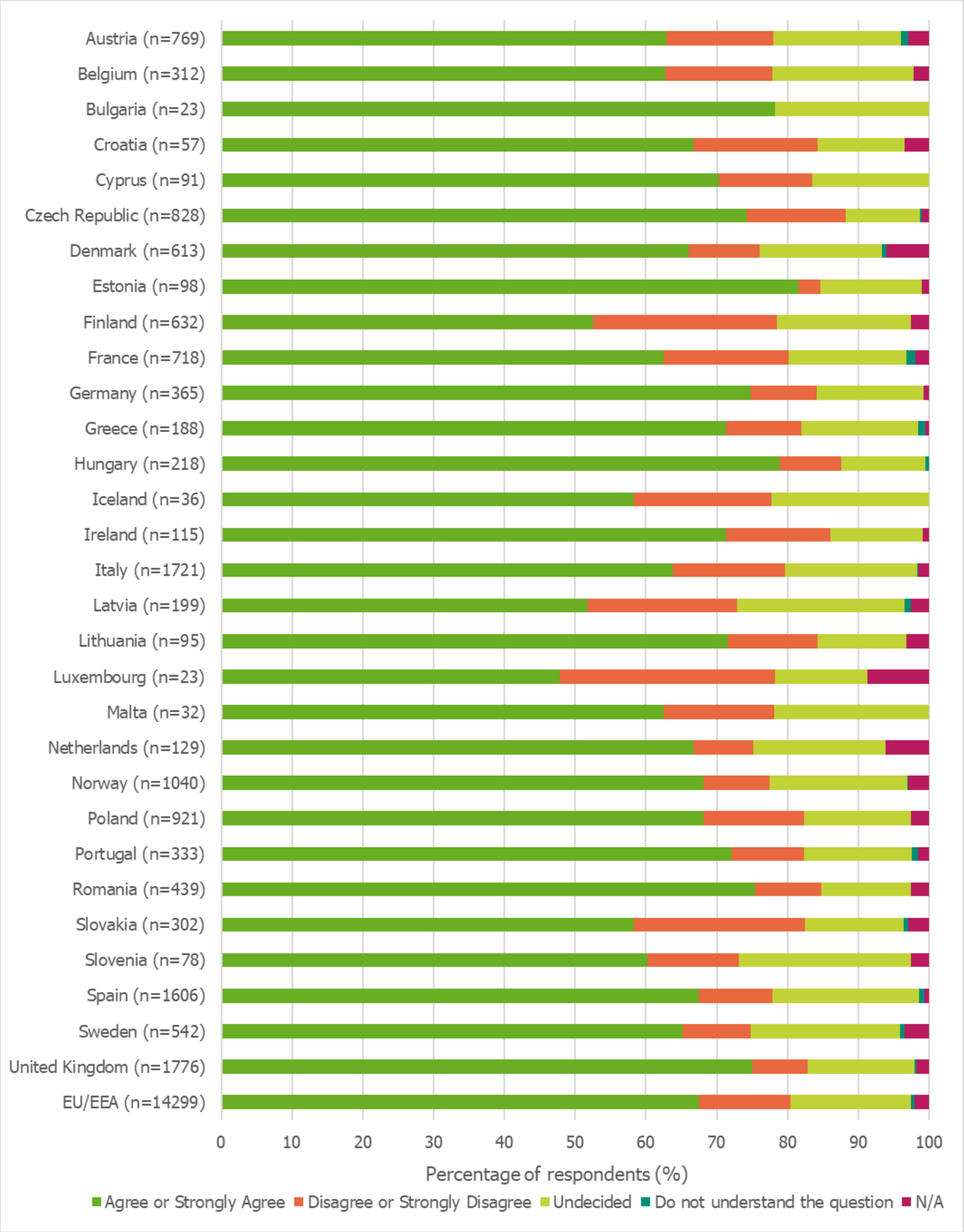

Supplementary Figure 6: Percentage of respondents who agreed or disagreed there had been good promotion of prudent use of antibiotics use and antibiotic resistance in their country, by country, EU/EEA (n=15 405)

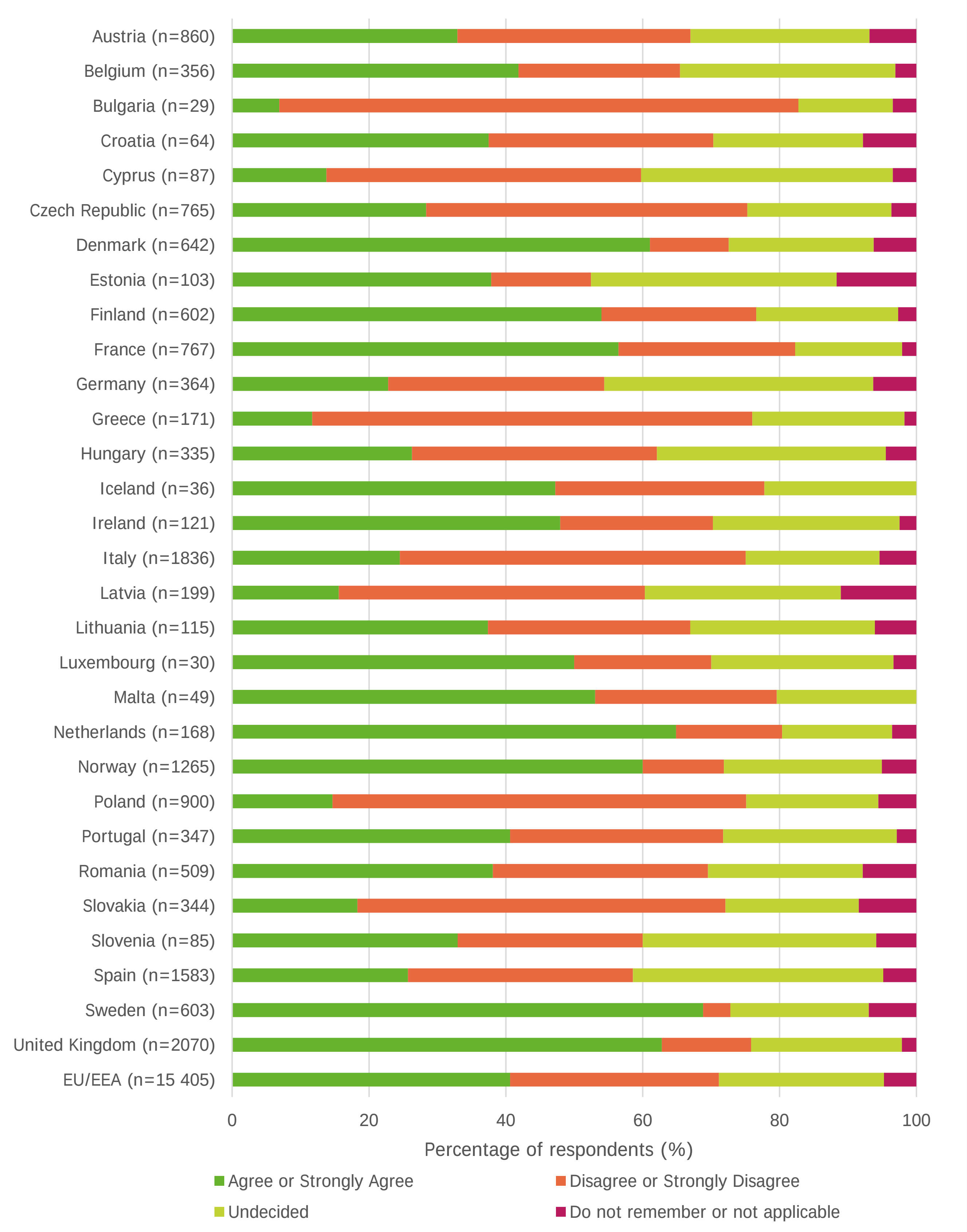

Supplementary Figure 7: Percentage of respondents that believe EAAD has been effective/ineffective in raising awareness about prudent use of antibiotics and antibiotic resistance in their country, by country

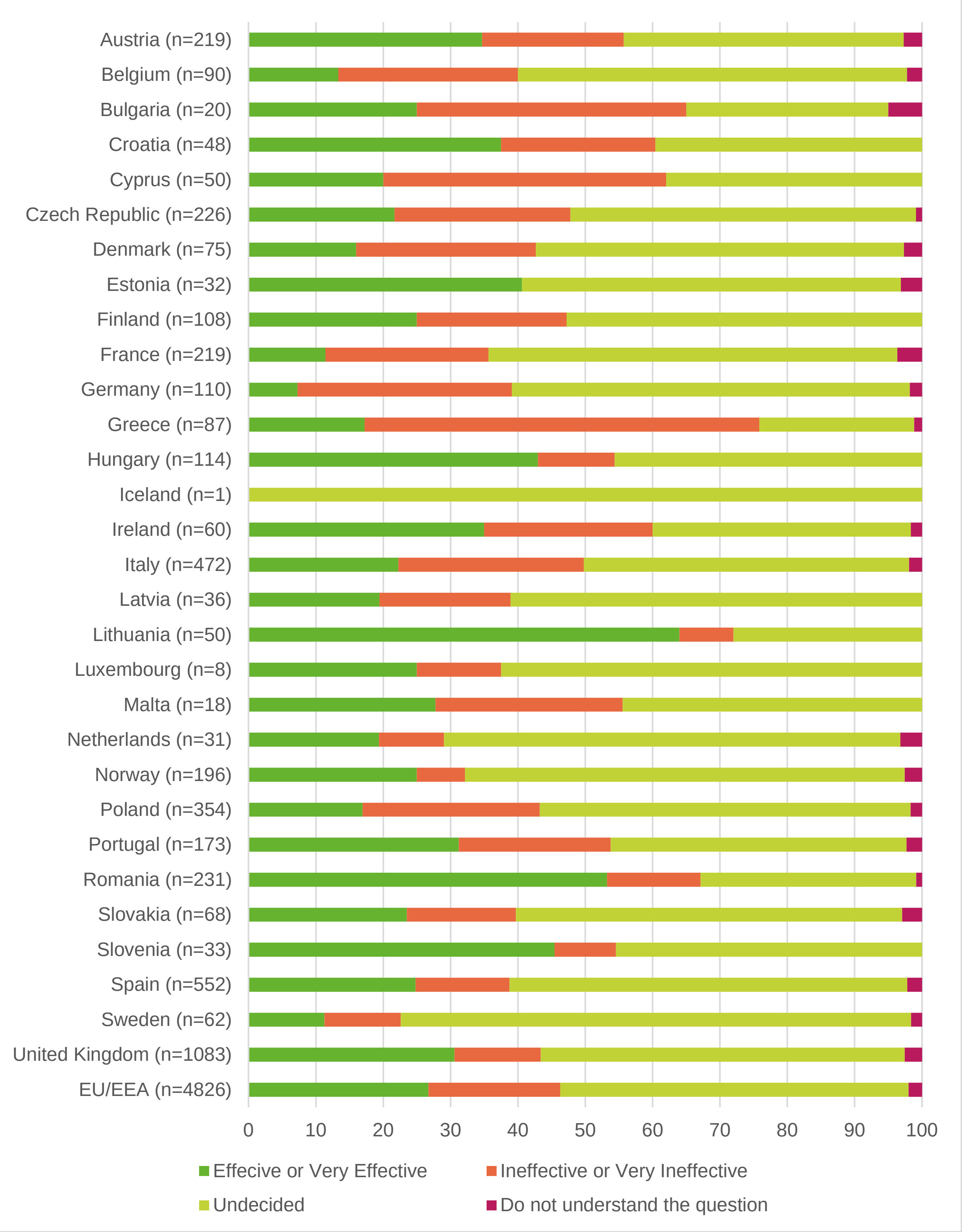

Supplementary Figure 8: Percentage of respondents who believe that WAAW has been effective/ineffective in raising awareness about prudent use of antibiotics and antibiotic resistance in their country, by country

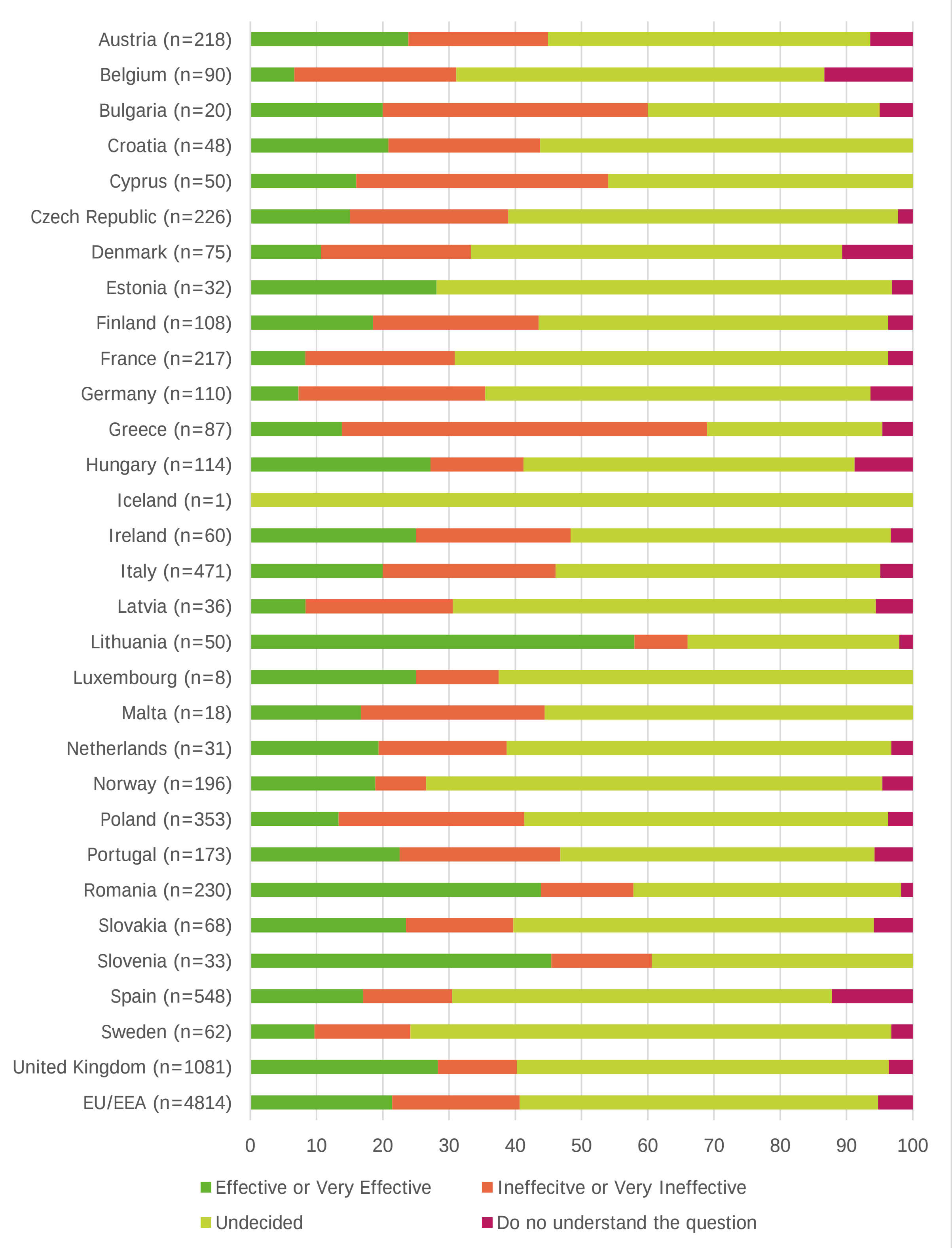

Supplementary Figure 9: Percentage of responding prescribers who frequently prescribed antibiotics due to the fear of patient deterioration or fear of complications during the last one week, by country, EU/EEA (n=6 508)

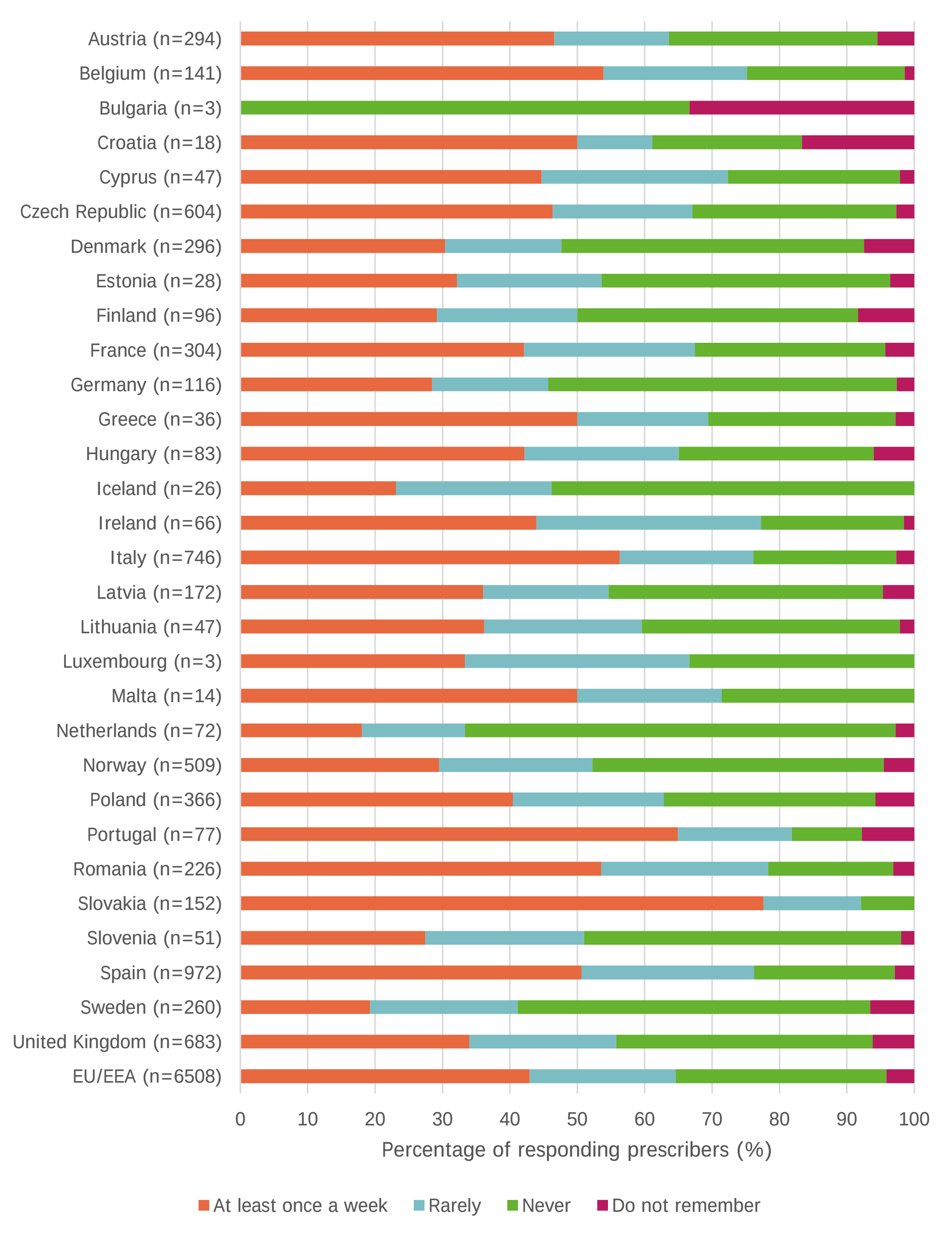

Supplementary Table 8: Comparison of prescriber behaviour on drivers for initiating prescriptions, by setting

Question	Setting	At least once a week, at least once a day, more than once a day, more than once a week or rarely n (%)	Never n (%)	OR (95% CI)	P- value
How often did the fear of patient deterioration or fear of complications lead you to prescribe antibiotics during the last one week? (n=6 508)	Hospital (n=2 901)	1 869 (64.4)	1 032 (35.6)	0.80 (0.71-0.90)	0.0003
	Community (n=1 996)	1 384 (69.3)	612 (30.7)		
How often did you prescribe antibiotics in situations in which it is impossible for you to conduct a follow-up of the patient during the last one week? (n=6 499)	Hospital (n=2 866)	1 135 (39.6)	1 731 (60.4)	0.95 (0.84-1.07)	0.4062
	Community (n=1 961)	800 (40.8)	1 161 (59.2)		
How often did you prescribe an antibiotic because you were uncertain about the diagnosis of infection during the last one week? (n=6 498)	Hospital (n=2 905)	1 454 (50.1)	1 451 (49.9)	0.97 (0.86-1.08)	0.5689
	Community (n=1 987)	1 011 (50.9)	976 (49.1)		
How often would you have preferred not to prescribe an antibiotic but were not able during the last one week? (n=6 511)	Hospital (n=2 834)	1 386 (48.9)	1 448 (51.1)	0.63 (0.56-0.71)	<0.0001
	Community (n=1 967)	1 185 (60.2)	782 (39.8)		
How often did you prescribe antibiotics because it took less time than to explain the reason why they are not indicated during the last one week? (n=6 507)	Hospital (n=2 917)	530 (18.2)	2 387 (81.8)	0.58 (0.51-0.66)	<0.0001
	Community (n=1 989)	551 (27.7)	1 438 (72.3)		
How often did you prescribe an antibiotic to maintain the relationship with the patient during the last one week? (n=6 498)	Hospital (n=2 886)	365 (12.6)	2 521 (87.4)	0.51 (0.44-0.60)	<0.0001
	Community (n= 1 973)	434 (22.0)	1 539 (78.0)		

Supplementary Table 9: Percentage of responding prescribers who agreed or disagreed that they felt supported to not prescribe antibiotics when they are not necessary, by country, EU/EEA (n=6 517)

Country	N	Agree or strongly agree (%)	Disagree or strongly disagree (%)	Undecided (%)	Do not know (%)
Austria	295	63	16	19	2
Belgium	141	70	13	16	1
Bulgaria	3	100	0	0	0
Croatia	18	22	50	22	6
Cyprus	47	64	13	23	0
Czech republic	606	50	21	23	6
Denmark	297	81	8	10	1
Estonia	28	71	7	18	4
Finland	96	72	11	15	2
France	305	64	19	15	2
Germany	116	59	22	17	1
Greece	36	44	28	28	0
Hungary	83	67	14	18	0
Iceland	26	58	15	23	4
Ireland	66	45	29	24	2
Italy	746	78	12	9	1
Latvia	172	63	15	19	3
Lithuania	47	81	6	13	0
Luxembourg	3	67	0	33	0
Malta	14	57	36	7	0
Netherlands	72	75	7	15	3
Norway	511	82	6	11	1
Poland	365	58	28	13	1
Portugal	77	66	14	19	0
Romania	226	75	15	9	2
Slovakia	152	51	32	16	1
Slovenia	51	80	4	10	6
Spain	972	52	20	27	1
Sweden	261	85	4	9	2
United kingdom	685	74	8	17	0
**EU/EEA**	**6 517**	**66**	**15**	**17**	**2**

Supplementary Table 10: Percentage of responding prescribers who agreed or disagreed that they felt supported to not prescribe antibiotics when they are not necessary, by professional setting, EU/EEA (n=6 517)

Setting	N	Agree or strongly agree	Disagree or strongly disagree	Undecided	Do not know
Community	2060	65	15	18	1
Governmental organisation	60	65	10	17	8
Hospital	3049	70	14	15	1
Industry	96	65	22	14	0
Long-term care facility	450	57	19	22	2
Not specified	21	57	29	14	0
Other	30	70	13	13	3
Pharmacy	20	65	25	10	0
Professional body	125	60	19	17	4
Public Health institute	176	66	15	17	2
University	88	80	7	11	2
Unknown	342	55	25	19	2
**EU/EEA**	**6 517**	**66**	**15**	**17**	**2**
